# The future is present in the past: A meta‐analysis on the longitudinal associations of parent–adolescent relationships with peer and romantic relationships

**DOI:** 10.1111/cdev.13849

**Published:** 2022-08-25

**Authors:** Susanne Schulz, Stefanie Nelemans, Hana Hadiwijaya, Theo Klimstra, Elisabetta Crocetti, Susan Branje, Wim Meeus

**Affiliations:** ^1^ Youth and Family Utrecht University Utrecht The Netherlands; ^2^ Department of Developmental Psychology Tilburg University Tilburg The Netherlands; ^3^ Department of Psychology University of Bologna Bologna Italy

## Abstract

Positive peer and romantic relationships are crucial for adolescents' positive adjustment and relationships with parents lay the foundation for these relationships. This longitudinal meta‐analysis examined how parent–adolescent relationships continue into later peer and romantic relationships. Included longitudinal studies (*k* = 54 involving peer relationships, *k* = 38 involving romantic relationships) contained demographically diverse samples from predominantly Western cultural contexts. Multilevel meta‐regressions indicated that supportive and negative parent–adolescent relationships were associated with supportive and negative future peer and romantic relationships. Meta‐analytic structural equation modeling (*k* = 54) indicated that supportive parent–adolescent relationships unidirectionally predicted supportive and negative peer relationships, while negative parent–adolescent relationships were bidirectionally associated with supportive and negative peer relationships. Maintaining mutually supportive relationships with parents may help adolescents to develop positive social relationships.

**Abbreviations**
MASEM: meta‐analytic structural equation modeling
OSMASEM: one‐stage meta‐analytic structural equation modeling

Adolescence is a crucial developmental period that is marked by rapid physical, cognitive, and social–emotional changes (Dahl et al., 2018). Achieving autonomy, gaining more egalitarian relationships with parents, as well as forming and maintaining high‐quality relationships with peers and romantic partners are among the most salient developmental tasks during this period. As adolescents navigate through changing social demands, their social relationships undergo major modifications and maturation. While parent–adolescent relationships become more egalitarian and reciprocal (Branje, 2018), peer and romantic relationships become increasingly intimate, committed, and mutually responsive (Bagwell & Bukowski, 2018; Furman & Buhrmester, 1992; Lantagne & Furman, 2017). Maintaining positive relationships with parents throughout adolescence is key to establishing positive relationships with other social partners. Social interactional and social cognitive theories (Baldwin, 1992; Bandura, 1977; Furman & Collibee, 2018; Hartup, 1979; Kaufman et al., 2020) commonly emphasize how the family context shapes later social relationships such as peer and romantic relationships. If adolescents fail to form positive relationships with peers and romantic partners, they are at risk for health and adjustment problems, such as increased mortality, loneliness, and depression (Cohen, 2004; Schwartz‐Mette et al., 2020). The role of the parent–adolescent relationship in shaping adolescents' peer and romantic relationships is thus also important for their general functioning.

Research supports theoretical perspectives by demonstrating that adolescents who have more positive parent–adolescent relationships indeed have relatively more positive peer and romantic relationships in adolescence and young adulthood (see Meeus, 2016 for an overview). However, large heterogeneity in effects sizes and assessed relationship dimensions at different ages renders it difficult to draw coherent conclusions about the strength of these associations and how they might differ by age. Furthermore, previous research mainly focused on unidirectional associations from parent–adolescent to future peer or romantic relationships, ignoring potential reversed associations from peer or romantic relationships to parent–adolescent relationships that might gain particular importance during adolescence. By synthesizing available information from longitudinal samples, the current meta‐analysis aims to (1) examine how interindividual differences in core dimensions of parent–adolescent relationships predict and precede subsequent interindividual differences in peer and romantic relationships, (2) examine potential bidirectional associations between parent–adolescent and other social relationships, and (3) investigate how these associations differ by age.

## Parent–adolescent relationships as foundation for future social relationships

Throughout the lifespan, people engage in relationships with various social partners that fulfill different functions. The very first relationships that children form are those with parents, which are involuntary, closed, and hierarchical by nature (Laursen & Bukowski, 1997). Lifespan and “social mold” theories propose that parent–child relationships influence not only the child's behavioral, emotional, and cognitive development, but also the child's social development (Hartup, 1979), including the formation and development of social relationships that are particularly crucial during adolescence. Relationships with parents at this time continue to affect social developmental processes (Ali et al., 2019; Collins & Laursen, 2004), potentially even more so as relationships with peers and romantic partners become increasingly important and acquire more mature relational functions.

Contrary to parent–adolescent relationships, peer relationships are voluntary, egalitarian, and temporary in nature (Laursen & Bukowski, 1997; Laursen & Veenstra, 2021). These relationships prepare adolescents for interactions among equals and the roles and expectations of adulthood. Early romantic relationships are similar to peer relationships in that both satisfy the adolescent's need for companionship (Shaver & Hazan, 1988). Toward late adolescence, peer and romantic relationships grow more intimate, and romantic relationships begin to take over relationship functions from parent–child relationships (Hazan & Shaver, 1987; Lantagne & Furman, 2017; Selfhout et al., 2009; Selman, 1989). As peer and romantic relationships resemble parent–adolescent relationships in their characteristics and functions, parent–adolescent relationships are suggested to provide a direct foundation for these developing social relationships.

### Theoretical rationale

In relationships with peers and romantic partners, adolescents apply interaction patterns that reflect relationships with their parents. Such continuity of relationship behaviors is often attributed to internal cognitive models or schemas that adolescents acquire in relationships with parents (Burks & Parke, 1996; Furman & Collibee, 2018). Schemas guide adolescents' social behavior by helping them to understand social cues and form expectations about others' behaviors, and are thus ultimately generalized to other relationships (Baldwin, 1992; Dodge & Pettit, 2003). Additionally, adolescents observe and enact early interactions with parents and model these learned behaviors in future interactions with peers or romantic partners (Bandura, 1977). According to social systems theory (e.g., Hartup, 1979), transfer from one social relationship to another also results from the interdependence between different social systems in which adolescents are embedded. Within the family system, parent–adolescent relationships provide the foundation that allows adolescents to explore their peer environment (Bronfenbrenner & Morris, 2006). As social systems are interrelated, the dynamics that occur within one system, such as structures, roles, patterns, or power relations, are therefore thought to spill over from one relationship to another (Erel & Burman, 1995). In that way, changes in the parent–adolescent system, which are fundamental during adolescence, can carry over to other relational systems with peers through modified emotions or behaviors (Benson et al., 2006; Kaufman et al., 2020). Spillover effects from relationships with parents to peer or romantic relationships may be particularly relevant in adolescence as this period is often considered a sensitive period for social learning (Dahl et al., 2018; Laursen & Veenstra, 2021).

Internal schemas are based on aspects of relationship quality that can be categorized into broad dimensions of relationships. Three core dimensions that become particularly important during adolescence when social competence and understanding increase are support, control, and negative interaction (Baumrind, 1991; Furman & Buhrmester, 1992; Furman & Collibee, 2018). Supportive parent–adolescent relationships, including aspects such as warmth, responsiveness, and nurturance, help adolescents to acquire positive relationship schemas, to develop prosocial, caring attitudes (Eisenberg et al., 2015), and to build interpersonal competence (Conger et al., 2000). They thus provide a positive foundation from which adolescents can explore other relationships and meet new relationship partners with trust and affection (Collins & Laursen, 2004). As friends become increasingly important sources of support during adolescence (Bagwell & Bukowski, 2018; Blos, 1967), adolescents are likely to generalize positive cognitive models and behaviors to peer and romantic relationships, which facilitates supportive social relationships (Rubin et al., 2015).

Similarly, individuation theory (Fousiani et al., 2014; Youniss & Smollar, 1985) posits that as adolescents strive for autonomy, optimal social development requires maintaining both high levels of support and increasingly lower levels of control in parent–adolescent relationships. Equality and independence become important features of social relationships as they promote adolescent well‐being and self‐validation (Bukowski et al., 2011). Negotiating and balancing control in parent–adolescent relationships are therefore important practice grounds for future egalitarian relationships with peers and romantic partners. Unbalanced controlling relationships, characterized by dominance, power, and lack of autonomy, may predict internalizing and externalizing problem behaviors (Pinquart, 2017a,b).

Negative interaction includes aspects such as conflict and hostility, but also physical and emotional abuse. While conflict interactions allow adolescents to integrate different expectations and can thus promote cognitive development and well‐being through negotiation and self‐reflection, frequent and intense negative interactions can strain social bonds and result in poor developmental and health outcomes (Laursen & Hafen, 2010). These processes may increase adolescents' aggressive behavior and decrease their interpersonal competence, which in turn increase negative interaction in later relationships with peers and partners (Conger et al., 2000; Patterson, 1982; Rubin et al., 2015).

### Bidirectional effects

Theoretical models on the continuity of social relationships are often based on “social mold” models (Collins & Sroufe, 1999; Furman & Collins, 2009; Hartup, 1979) and assume unidirectional pathways from parents to adolescents (i.e., parent‐to‐adolescent effects), in which experiences with parents provide the foundation for later experiences with peers and romantic partners. During adolescence, children strive for more independence from parents and spend more time outside the family with friends and romantic partners (Branje et al., 2021). As peer and romantic relationships become increasingly important, they can also play a role in transforming parent–adolescent relationships. Transactional models (Bell, 1968; Sameroff, 2009) propose that children actively shape their environment and are thus not only influenced by their parents, but also influence their parents. This may apply even more so in adolescence when major developmental changes such as increasing social understanding, competence, and independence motivate changes in social relationships (Branje et al., 2021; Lantagne & Furman, 2017; Laursen & Bukowski, 1997). In maturing peer and romantic relationships, adolescents practice equality and autonomy interactions, which allow them to acquire new relational schemas and interpersonal skills that are generalized to other relationships (Graziano, 1984). The need for similarity with peers during this period further renders adolescents particularly susceptible to peer influence and the adaptation of their own behaviors and attitudes to those of their peers and romantic partners (Laursen & Veenstra, 2021). In line with the spillover hypothesis (Erel & Burman, 1995), peer or romantic relationships may also affect adolescents' general emotional states through which interaction patterns can carry over from one relational system to another, resulting in bidirectional associations between parent–adolescent and peer or romantic relationships.

### Empirical evidence

Research supports the theoretical continuity of parent–child relationship quality into peer and romantic relationships. Evidence from meta‐analyses and reviews found small to moderate positive associations between early mother–child attachment and peer relationships in childhood (Pallini et al., 2014; Schneider et al., 2001), as well as moderate positive associations between parent and peer attachment in adolescence (Gorrese & Ruggieri, 2012). These findings are extended by studies reporting longitudinal associations between parent–adolescent relationships and peer or romantic relationships in adolescence and emerging adulthood (for a narrative review, see Meeus, 2016). For the extreme negative end of the relationship quality distribution, meta‐analytic evidence showed links between violent parent–child relationships and violent romantic relationships in adulthood through witnessing interparental violence and experiencing parental violence (Smith‐Marek et al., 2015; Stith et al., 2000).

Most previous meta‐analytical evidence focused on perceived attachment or the affective quality of relationships, and not on how the core dimensions of support, control, and negative interaction are transferred from parent–adolescent relationships to peer or romantic relationships in adolescence and emerging adulthood. Furthermore, these meta‐analyses focused solely on transfer of similar features of relationship dimensions and thus do not allow for conclusions about whether these aspects also generalize to other relationship dimensions. Perspectives on family socialization (Grusec & Davidov, 2010) indicate that each relationship dimension holds distinct functions and thus, associations might be strongest among similar relationship dimensions with parents and peers or partners. Other perspectives propose that cognitive models and representations are relatively global and that specific relationship aspects thus affect relationship quality more generically (Conger et al., 2000; Overall et al., 2003; Youniss & Smollar, 1985). Hence, specific relationship dimensions might influence not only the same, but also other relationship dimensions (e.g., highly supportive parent–adolescent relationships might not only predict more supportive, but also less negative peer or romantic relationships). The few studies assessing continuity between parent–adolescent and other social relationships across different relationship dimensions remain inconsistent. While some research suggests associations of more negative parent–adolescent relationships with less supportive future peer (Gayman et al., 2010) and romantic relationships (Slominski et al., 2011), others failed to replicate these findings (Andrews et al., 2000). The current study extends previous meta‐analyses by examining how the three core relationship dimensions support, negative interaction, and control in parent–adolescent relationships continue into later peer and romantic relationships, both within as well as across relationship dimensions.

Furthermore, while previous studies mainly focused on relationships with parents during (early) childhood, this meta‐analysis focuses on relationships with parents during adolescence and emerging adulthood. Adolescence is a particularly important developmental period during which social relationships undergo major changes. As relationships with parents mature and become more similar to relationships with peer and romantic partners, aspects of these changing relationships may be more likely to transfer to peer and romantic relationships than aspects of earlier parent–child relationships (Schneider et al., 2001).

Finally, most studies to date focused on theoretical conceptions emphasizing that parent–adolescent relationships constitute the foundation of later social relationships in adolescence and emerging adulthood and thus mainly focused on unidirectional associations between parent–adolescent and peer or romantic relationships. However, they often combined cross‐sectional and longitudinal evidence, which does not allow for conclusions on directionality and temporal order of these associations. Moreover, changes in social relationships and the increasing importance of peers and romantic partners during adolescence suggest that peer and romantic relationships might influence and thus further transform parent–adolescent relationships. Initial research supports bidirectional links between peer and parent–adolescent relationships in adolescence, with associations between support from parents to peers decreasing from early to late adolescence and associations between negative interaction from parents to peers increasing (De Goede et al., 2009). Another study, however, failed to detect reversed associations between support from parents and support from peers in adolescence (Luyckx et al., 2012). Apart from these few inconsistent findings, longitudinal studies to date rarely emphasized the potential lagged effects of peer and romantic relationships on later parent–adolescent relationships. Including such reversed associations is crucial to disentangle whether the associations of parent–adolescent relationships with peer or romantic relationships in adolescence are stronger for parent‐to‐adolescent or adolescent‐to‐parent effects. In this meta‐analysis, we, therefore, aimed to examine whether associations between parent–adolescent and later adolescent or emerging adult peer and romantic relationships are unidirectional, and thus provide support for parents as foundation for social relationships, or whether they are bidirectional, and thus provide support for transactional processes.

## Moderators of the associations between parent–adolescent and peer and romantic relationships

### Time effects

As the time interval between measurement occasions increases, the associations of parent–adolescent relationships with peer and romantic relationships are likely to become smaller. If parent–adolescent relationships are measured in close proximity to peer or romantic relationships, there may be fewer intervening variables and thus parent–adolescent relationships may be more strongly associated with peer or romantic relationships. If they are measured further apart, events or changes in parent–adolescent relationships might occur that alter the quality of peer and romantic relationships, and weaken the associations with parent–adolescent relationships. *Revisionist* perspectives support this idea and propose that current experiences decrease the effects of previous relationship experiences with parents (Fraley & Roisman, 2015).

Alternatively, previous relationships with parents might provide long‐lasting working models that continue to shape adolescent development (Sroufe et al., 1990). This perspective suggests that the effects of previous parent–adolescent relationships are *enduring*, indicating that the associations with peer or romantic relationships stabilize over time (Fraley & Roisman, 2015). As both revisionist and enduring perspectives emphasize the importance of time in how parent–adolescent relationships continue into peer and romantic relationships, this meta‐analysis accounts for the potentially varying effects of time in the continuity of social relationships.

### Adolescent age

As autonomy and voluntary interactions gain more importance, the main focus of adolescent relationships gradually shifts from parents to peers and romantic partners (e.g., Branje et al., 2021). Although parents remain relevant sources for support, adolescents are more likely to turn to peers or romantic partners for support (Bagwell & Bukowski, 2018; Youniss & Smollar, 1985). As adolescents gradually become more autonomous from their parents, parents are less involved in adolescent lives. Their relationships with adolescents might thus become less influential in predicting future peer and romantic relationships over time. Peer and romantic relationships, however, grow more important as adolescents get older and provide optimal grounds to apply interaction patterns based on egalitarian principles (Lantagne & Furman, 2017; Laursen & Bukowski, 1997). As such, they might also become more influential in predicting later relationships with parents over time.

On the other hand, parent–adolescent relationships become more reciprocal and egalitarian throughout adolescence (Eccles et al., 1993; Hadiwijaya et al., 2017). As they realign toward greater horizontality, they more closely resemble peer and romantic relationships. Similarly, processes and functions that are specific to romantic relationships only emerge later in adolescence and young adulthood (Furman & Collins, 2009). Particularly early romantic relationships often resemble close peer relationships more than intimate sexual relationships. As peer and romantic relationships become more important and more similar to parent–adolescent relationships over time, the links between parent–adolescent and peer or romantic relationships might also become stronger with age.

Longitudinal studies generally support associations of parent–adolescent relationships with later peer or romantic relationships in early adolescence (Kochendorfer & Kerns, 2017; Rice & Mulkeen, 1995), mid‐adolescence (Giordano et al., 1998; Kaufman‐Parks et al., 2018), and late adolescence or early adulthood (De Goede et al., 2009; Slominski et al., 2011). However, these studies do not permit conclusions about whether and how these associations change over time. While research on how parent–adolescent relationships continue into peer and romantic relationships over time remains scarce, initial findings are in line with separation theories, suggesting that these associations decrease for support, but not for negative interaction or control (e.g., De Goede et al., 2009). Contrasting findings are in line with realignment theories, indicating that the associations between parent–adolescent relationships and romantic relationships increase as adolescents grow older (Kaufman‐Parks et al., 2018; Meeus et al., 2007). Other research, however, did not detect any age effects (De Goede et al., 2012). Inconsistent findings, variation in time lags between measurements, and the inclusion of different age groups across studies render it difficult to draw coherent conclusions about the strength of associations between parent–adolescent and peer or romantic relationships across adolescence. This meta‐analysis addresses these issues by investigating how time lag between measurements and adolescent age might moderate the effects of parent–adolescent relationships on peer and romantic relationships. Investigating temporal differences will provide insights into short‐ or long‐term effects of parent–adolescent relationship quality.

## The present study

**FIGURE 1:**
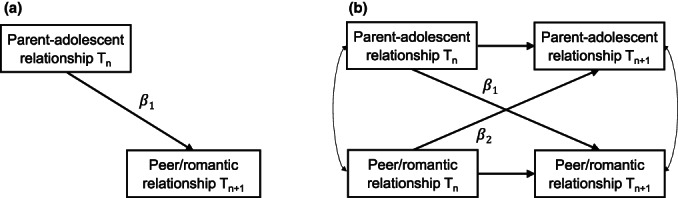
Graphical representation of the conceptual three‐level regression model (a) in which *β*1 denotes the parent‐to‐peer regression path, and the cross‐lagged panel model (b) in which *β*1 and *β*2 denote the parent‐to‐peer and peer‐to‐parent regression paths, respectively, controlling for autoregressive stability paths and concurrent correlations.

This longitudinal meta‐analysis aims to synthesize and expand previous heterogenous evidence on how core dimensions of parent–adolescent relationships continue into future peer and romantic relationships. This is needed because previous meta‐analyses often did not allow for conclusions on the temporal order of associations between parent–adolescent relationships and subsequent peer and romantic relationships, and longitudinal findings largely vary regarding the magnitude of associations between parent–adolescent and other social relationships, both within the same and particularly across different relationship dimensions. In the present meta‐analysis, we applied two distinct, but complementary statistical approaches to provide generalizations about the extent of relationship continuity across core dimensions of social relationships: First, we used multilevel meta‐analytic regressions (Cheung, 2014) to examine how support, negative interaction, and control in parent–adolescent relationships are related to support, negative interaction, and control in future peer and romantic relationships (see Figure 1a). This design allowed us to include all existing information within and across studies while accounting for multiple effect sizes from the same study. We expected that support, negative interaction, and control in parent–adolescent relationships continue into future peer and romantic relationships, within the same and across relationship dimensions.

Second, we used meta‐analytic structural equation modeling (MASEM; Jak, 2015) to investigate how support, negative interaction, and control in parent–adolescent relationships predict relative change in support, control, and negative interaction in future peer relationships, controlling for over‐time stability, concurrent correlations, and potential lagged associations of peer on parent–adolescent relationships (see Figure 1b). Unlike the multilevel approach, MASEM allowed us to model structural pathways. We expected that support, negative interaction, and control in parent–adolescent relationships predict peer relationships above and beyond over‐time stability, concurrent, and reversed correlations.

Based on contrasting theories and findings, it remains unclear how and to what extent the links between parent–adolescent relationships and future peer or romantic relationships change as measurements are further apart in time or as adolescents get older. We, therefore, examined whether the associations of parent–adolescent relationship quality with peer and romantic relationship quality vary depending on time interval between measurement occasions (i.e., short‐term vs. long‐term effects) and adolescent age. Due to the increasing importance and influence of peer and romantic relationships, potential reversed associations were expected to become stronger as adolescents got older. In addition, we explored other sample and study characteristics that may explain variation within and across studies.

## METHODS

The design, research aims, and hypotheses of this meta‐analysis were preregistered (https://www.crd.york.ac.uk/PROSPERO/display_record.php?RecordID=103492). All hypotheses were confirmatory in nature, except the analyses involving sample and study characteristics, which were explored as potential moderators.

### Eligibility criteria, data sources, and study selection

#### Eligibility criteria

Our literature search aimed to identify studies that examined longitudinal associations between parent–adolescent relationship quality and adolescents' peer or romantic relationship quality. We used three main criteria to select studies: First, selected studies included an adolescent or emerging adult sample at the earliest measurement. Studies were excluded if the initial sample at *T*_*n*_ included participants younger than 10 years or older than 25 years. Second, studies assessed relationship aspects for both parent–adolescent and peer or romantic relationships that correspond to core dimensions of relationship quality (i.e., support, negative interaction, or control). We excluded measures that did not focus specifically on parents, such as measures that focused on family more generally, which could include other family members, aspects that did not constitute relationship quality, such as peer affiliation or peer victimization, or aspects that we could not assign to core dimensions, such as general relationship quality (i.e., composite measures of various core components) or attachment. Studies that measured attachment in the traditional sense (e.g., secure or avoidant attachment) were excluded. Studies that used the term attachment to refer to aspects of relationship quality (e.g., closeness measured with the Inventory of Parent and Peer Attachment; Armsden & Greenberg, 1987) were included as measures of support. Retrospective reports on relationship quality with parents and peers or romantic partners that dated back to >1 year were excluded as they could introduce potential bias and distortions and thus, do not accurately reflect relationship quality at that time. Third, studies included a longitudinal study design in which parent–adolescent relationship assessments preceded peer and romantic relationship assessments in time. Lastly, we set no language restrictions and if needed, received help from native speakers to decode non‐English articles.

#### Literature search

Our strategy to identify relevant articles was fourfold: First, we searched for peer‐reviewed journal articles in the databases ERIC, PsycArticles, PsycInfo, Scopus, and Web of Science for all years until August 2018. We used a combination of search terms that reflected (1) an adolescent or young adult sample, (2) relationship dimensions with parents, peers, or romantic partners, and (3) a longitudinal study design. The exact search strings for each of the databases are provided in Supporting Information. Second, we searched the table of contents of the top 10 journals deemed most likely to publish studies on adolescent interpersonal relationships. Specifically, we manually searched for articles in the most recent issues and online first or early view sections of the top 10 journals identified by Web of Science and SCOPUS as having the most records concerning our search terms. Third, we searched for articles by examining the reference sections of relevant review articles (e.g., Meeus, 2016) and of all articles included in current meta‐analysis. Finally, we searched for unpublished materials, such as dissertations, conference proceedings, policy documents, and other reports, in 15 databases (see Supporting Information for more details on the searched databases and screening process).

Our search strategy for published studies yielded 6753 unique journal articles (see Figure S1). Two subsequent screening procedures of titles and abstracts as well as full texts by the first and third author resulted in an inclusion of 145 articles. Interrater reliabilities on 25% of all studies were good (*K* = .91 and *K* = .89, respectively). Diverging assessments were discussed among the co‐authors until consensus was reached. More than half of the studies (59%) did not report the required effect sizes or reported effect sizes derived from multivariate analyses. In these cases, we contacted the authors to provide the missing zero‐order correlations between continuous study variables, biserial correlations between continuous and dichotomous study variables, and polychoric correlations between dichotomous study variables. If necessary, reminders were sent after 2 weeks. Of all 97 authors contacted, 47 provided the requested effect sizes, 22 could not provide the requested correlations, and 28 did not reply to our request. This led to an exclusion of 42 studies.

Our search strategy for unpublished materials yielded an additional 1381 documents (see Figure S1). Two subsequent screening procedures of titles and abstracts as well as full texts by the first author and two graduate students resulted in an inclusion of 67 documents. Interrater reliabilities on 10% of all studies were good (*K* = .81–.92 and *K =* .88, respectively). Of these 67 documents, 27 were excluded because the relevant correlations had already been published. For studies that did not report the required effect sizes (*n* = 11), we contacted the authors using the same strategy as reported above for journal articles. One study was excluded because no up‐to‐date author information could be retrieved. Of all 10 authors contacted, three provided the requested effect sizes, but two of those studies had to be excluded because the relationship constructs combined several relationship dimensions. Seven authors did not reply to our request or could not provide the requested correlations. Hence, nine studies were excluded. All included unpublished studies were dissertations.

As common in longitudinal research, we identified several studies that used the same datasets as other included studies (*k* = 65, including 42 published and 23 unpublished studies, on 12 different datasets). To ensure that we did not include overlapping data, we excluded all duplicated information based on a three‐fold strategy: First, of all studies that used the same dataset, we included the study with the earliest assessment, unless another study on the same dataset used a sample size >25% of the previous study. Second, if two studies used the same first assessment and sample size, we included the study that contributed the most effect sizes. If the same data were used in published and unpublished materials, we included published studies to increase methodological soundness. Third, if other studies on the same dataset contributed effect sizes that were not assessed in the first study (e.g., additional relationship dimensions or waves), we also included those unique effect sizes. This strategy led to a final inclusion of 87 studies on 80 unique datasets with 100 independent samples, of which 54 studies involved peer outcomes and 38 studies involved romantic outcomes.

### Data extraction

A structured coding manual guided the coding procedures. To obtain estimates of coder reliability, the first and third author coded 25% of all studies. The interrater reliability was good to perfect (*K* = .98 to *K* = 1.0). Diverging assessments were discussed among co‐authors until consensus was reached. After obtaining agreement, all remaining studies were coded by either one of the two first authors. Unpublished materials were coded by two graduate students (*K* = 1.0 on 50% of all studies). More information on the coding procedures and decisions, including interrater reliabilities for each coded category, can be found in Supporting Information. Cases for which we could not clearly retrieve the required information were coded as missing. Table S1 displays the sample characteristics of all studies for peer (1A) and romantic outcomes (1B).

#### Publication

We coded publication status, the year of publication, the journal impact factor, and the associated quartile ranking (i.e., higher impact factors and lower quartile rankings, e.g., Quartile 1, indicate higher quality journals) based on the year of publication. In cases in which impact factor and ranking were unknown, we used the impact factor and quartile of the year closest to the year of publication.

#### Study procedures

We coded information regarding recruitment and waves. Recruitment characteristics included geographical region of the sample (i.e., North America, Western Europe, South America, Eastern Europe, Asia, or Oceania), recruitment location (i.e., local or national), recruitment strategy (i.e., convenience sampling, selective sampling, or random sampling), and percentage of attrition across waves. Wave characteristics included number of waves measuring parent–adolescent relationship and peer or romantic relationship, respectively, and time lag between measures.

#### Sample

Sample characteristics at the assessment of the parent–adolescent relationship at time point *T*_*n*_ included total sample size, mean age of adolescents, percentage of adolescent boys, percentage of racial‐ethnic minorities within the national context, percentage of participants living with both or single parents, and type of population (i.e., community, marginalized, or clinical). If studies reported an age range of the participating adolescents, we estimated the (weighted) average. If studies reported only the grade level(s) of the participating adolescents but no age range (*k* = 7 for peer, *k* = 9 for romantic relationships), we estimated age based on the country's general age range per grade level. Marginalized population in this study refers to populations with low socioeconomic status or racial‐ethnic minorities as specified per study. Sample characteristics at the assessment of the peer or romantic relationship at *T*_*n*+1_ included sample size, mean age of adolescents, degree of friendship (i.e., peers, close friend, or best friend), type of romantic sexuality (i.e., straight, sexual minorities only, or all sexual orientations included), and whether the same peers or romantic partners were assessed across waves.

#### Measures

Measure characteristics regarding the relationship constructs (i.e., with parent, peer, or romantic partner) included type of parent–adolescent relationship (i.e., both parents, mother, or father), number of relationship constructs, relationship dimension (i.e., support, negative interaction, or control), shared informant (i.e., whether the same assessment method was used for parent–adolescent and peer or romantic relationship, such as same reporter), instrument (i.e., type of questionnaire or task), and the interrater reliability of the measure. Relationship aspects such as warmth, responsiveness, nurturance, or prosocial behavior were classified as support; relationship aspects such as conflicts, arguments, hostility, or abuse were classified as negative interaction; and relationship aspects such as authority, dominance, power, and lack of autonomy were classified as control. To avoid additional levels of dependency in our effect sizes, we used weighted means to average multiple effect sizes on the same wave and construct across different types of parent–adolescent relationship (i.e., mother–adolescent and father–adolescent relationship), different reporters (e.g., adolescent reporter, rater), or different instruments.

#### Effect sizes

We used Pearson's correlations of continuous relationship scores with at least one time lag between assessments to operationalize the longitudinal associations between parent–adolescent relationships and peer or romantic relationships. If studies provided correlations for more than two waves, we recorded all available correlation coefficients. If available, we recorded all stability coefficients between parent–adolescent and peer relationship, all concurrent correlation coefficients between parent–adolescent and peer relationship at *T*_*n*_ and *T*_*n*+1_, and all bidirectional correlation coefficients between peer relationship at *T*_*n*_ and parent–adolescent relationship at *T*_*n*+1_.

### Statistical analyses

All statistical analyses were performed using the package metaSEM (Cheung, 2015) for the software program R 3.5.1 (R Core Team, 2019), following the approaches outlined by Cheung (2014) for multilevel meta‐analyses and Jak (2015) for MASEM analyses. Random‐effect models were used for all analyses to account for heterogeneity among studies. While fixed‐effects meta‐analysis assumes one single mean population effect size and identical study conditions, which is hardly realistic, random effect models assume a distribution of the mean population effect size and take varying study conditions into account (Hedges & Vevea, 1998). For all analyses, we modeled the raw correlations as recommended for random effects models (Schulze, 2004). Parameter estimates were obtained with maximum likelihood estimation, and missing data were handled with full information maximum likelihood estimation. All standardized estimates can be interpreted in terms of effect size *r*, with values around .10 indicating small effects, values around .30 indicating intermediate effects, and values around .50 indicating strong effects (Bollen, 1989). As current statistical procedures do not yet allow the performance of structural equation modeling on multilevel meta‐analytic data, we conducted two complementary analyses to answer our research questions (see Figure 1).

#### Three‐level meta‐analyses

**FIGURE 2:**
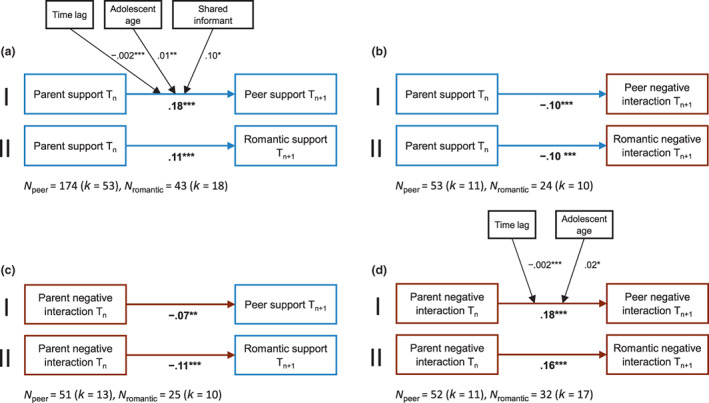
Main and significant moderation outcomes for three‐level meta‐regression analyses between supportive (a, b) and negative parent–adolescent relationships (c, d) and future peer (I) and romantic relationships (II). All effect sizes refer to standardized *β*s. ***p < .001, **p < .01, *p < .05.

Traditional meta‐analysis assumes that the effect sizes are statistically independent. However, most included studies reported multiple effect sizes (e.g., the same correlation was measured at different waves), which indicates dependence among these effect sizes. To include all available study information, we used a three‐level approach to account for dependent effect sizes. Specifically, the first level reflects the sampling variance around the estimated population effect size, the second level reflects the variance between effect sizes within studies, and the third level reflects the variance between effect sizes between studies (Cheung, 2014). To estimate how supportive, negative, and controlling parent–adolescent relationships are associated with supportive, negative, and controlling future peer relationships (see Figures 1a and 2‐I), we performed nine three‐level random‐effect regression analyses—three analyses for each parent–adolescent relationship dimension (i.e., support, negative interaction, and control), respectively. Similarly, to estimate how supportive, negative, and controlling parent–adolescent relationships are associated with supportive, negative, and controlling future romantic relationships (see Figures 1a and 2‐II), we performed nine additional three‐level random‐effect meta‐regression analyses.

#### Cross‐lagged MASEM analyses

**FIGURE 3:**
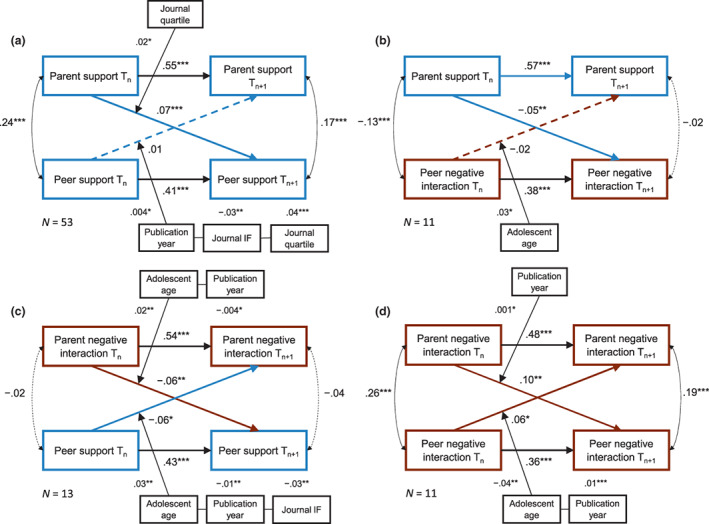
Main and significant moderation outcomes for meta‐analytic structural equation modeling analyses between supportive (a, b) and negative parent–adolescent relationships (c, d) and future peer relationships; Journal IF = journal impact factor. All effect sizes refer to standardized *β*s. ***p < .001, **p < .01, *p < .05.

To estimate how supportive, negative, and controlling parent–adolescent relationships predict future peer relationships over time (see Figures 1b and 3), we performed nine cross‐lagged MASEM analyses—three analyses for each parent–adolescent relationship component, respectively. Specifically, we added five additional paths to the model: (1) the stability of parent–adolescent relationship from *T*_*n*_ to *T*_*n*+1_, (2) the stability of peer relationship from *T*_*n*_ to *T*_*n*+1_, (3) the concurrent correlation of parent–adolescent and peer relationship at *T*_*n*_ (4) the concurrent correlation of parent–adolescent and peer relationship at *T*_*n*+1_, and (5) the reversed correlation from peer relationship at *T*_*n*_ to parent–adolescent relationship at *T*_*n*+1_. MASEM analyses do not allow for multiple effect sizes per study. Therefore, we selected only the first effect size per study for the analyses, as the first assessment point usually contained the largest sample size. To estimate the cross‐lagged models, we used the one‐stage MASEM approach (OSMASEM; Jak & Cheung, 2020). This approach is an extension of the conventional two‐stage approach that first pools correlations coefficients and then fits the structural model to the pooled correlation matrix (TSSEM; Cheung, 2014). In OSMASEM, the structural model is fitted directly on the observed correlation matrix, rather than the pooled correlation matrix. Both approaches result in the same parameter estimates and standard errors in models without moderators (Jak & Cheung, 2020). Unlike previous approaches, however, OSMASEM allows the modeling of continuous moderators on the structural paths while accounting for missing correlations.

#### Moderator analyses

Heterogeneity across effect sizes was assessed in all models, using the *Q*‐statistic and *I* ^2^ measure from Stage 1 of the two‐stage MASEM approach. The *Q*‐statistic evaluates whether there is substantial variability in the effect sizes across studies (*τ* ^2^ ≠ 0). The *I* ^2^ quantifies how much variation across studies is attributed to heterogeneity, with values >25%, >50%, and >75% representing small, moderate, and large heterogeneity, respectively (Higgins et al., 2003). We reported all effect sizes to two decimal places. For small moderation effects, we deviated from this rule if rounding to two decimal places would result in non‐informative, imprecise estimates.

If the analyses detected significant heterogeneity between effect sizes, we examined whether hypothesized moderators might explain this heterogeneity. As potential moderators were prespecified, we did not add all moderators successively to the model but conducted all moderator analyses separately to refrain from inflating the type II error rate and maximize statistical power. For both statistical procedures, we tested each moderator's unique contribution by adding them as predictors to the final models and calculating the proportion of explained variance (e.g., Cheung, 2014). $R_{\text{within}}^{2}$ and $R_{\text{between}}^{2}$ indicate how much estimated heterogeneity each predictor explained within (level 2) and between studies (level 3). However, this approach did not allow us to examine the unique effects of one moderator when accounting for other moderators. Hypothesized moderators were adolescent age and time between measurements. Additional moderators included type of population, shared informant, percentage males, percentage racial‐ethnic minority, publication status, publication year, journal impact factor, and journal quartile. Continuous moderators were centered on the mean.

#### Publication bias

Publication bias is a major concern in meta‐analyses as it may inflate the associations between two constructs, resulting in false conclusions. To date, most methods assessing publication bias suffer from serious limitations, particularly with regard to the small number of included studies and effect sizes (see van Aert et al., 2019 for a review). Furthermore, severe heterogeneity between studies prevents a clear detection of publication bias as it often results in an increase in false positives (Terrin et al., 2003). To get a more comprehensive picture of the potential presence and implication of publication bias in this study, we used two established methods. First, we examined funnel plots and conducted Egger's tests to estimate potential associations between effect sizes and their precision (i.e., small study effects). Asymmetrical funnel plots indicate the presence of small study effects. The Egger's test outperforms comparable methods and is the recommended method to assess publication bias in meta‐analyses (van Aert et al., 2019). However, the Egger's test often fails to produce reliable results in samples with <10 effect sizes due to insufficient power, resulting in an increase of false positives (Sterne et al., 2000; Terrin et al., 2003). Furthermore, it should be noted that asymmetrical funnel plots indicate small study effects, of which publication bias is only one possible cause (Egger et al., 1997). Second, we used the three‐parameter selection model (Iyengar & Greenhouse, 1988) to estimate effect sizes corrected for potential publication bias. This approach allows researchers to compare the unadjusted to bias‐adjusted models using likelihood ratio tests and outperformed other effect size correction methods in simulation studies (Carter et al., 2019; van Aert et al., 2019).

## RESULTS

Table S2 provides a general overview of all main and moderation results.

### Three‐level meta‐regressions on parent–adolescent and future peer and romantic relationships

#### Sample description

For peer outcomes, our search yielded *N* = 431 effect sizes from *k* = 54 independent studies with a total of 62 samples and 51,891participants. For romantic outcomes, our search yielded *N* = 147 effect sizes from *k* = 38 independent studies with a total of 43 samples and 18,763 participants. Further information regarding the number of effect sizes per sub‐analysis is depicted in Table S3A. The majority of studies sampled intact (72.0%), racial‐ethnic majority families (63.1%) from Western countries (96.0%) and assessed relationship quality using adolescent informants only (79.0%). Further study characteristics are depicted in Table S1A. Forest plots with all effect sizes, their corresponding confidence intervals, and interpretation are depicted in Supporting Information (Figures S2).

#### Peer outcomes

Due to the small number of studies assessing control in parent–adolescent or peer relationships (*k* = 2), these analyses were not interpreted (but see Table S4). For all remaining relationship dimensions, parent–adolescent relationship quality was significantly associated with later peer relationship quality (see Figure 2‐I; Table S3A). More supportive relationships with parents were associated with more supportive (*β* = .18, *p* < .001) and less negative (*β* = −.12, *p* < .001) subsequent relationships with peers. Similarly, more negative relationships with parents were associated with more negative (*β* = .18, *p* < .001) and less supportive subsequent relationships with peers (*β* = −.07, *p* = .002).

#### Test of moderators

All analyses revealed significant heterogeneity within and across studies (see Table S3A). Levels of heterogeneity were moderate to large between, but negligible to small within studies. Therefore, we conducted several analyses to examine whether theoretical moderators (i.e., time lag, adolescent age) and exploratory moderators (i.e., population, shared informant, percentage males, percentage racial‐ethnic minority, publication status, publication year, journal impact factor, and journal quartile) significantly explained these differences (see Table S5 for descriptive statistics on all moderators). Only few moderators (i.e., time lag, adolescent age, shared informant) significantly improved the model fit for some relationship dimensions (see Table S6). The results of these moderators are depicted in Table S7 (see also Figure 2‐I).

##### Time lag

Adding time lag as a moderator significantly improved the model fit for two of four analyses: If the measurements were closer together in time, supportive parent–adolescent relationships were more strongly associated with supportive peer relationships (*β* = −.002, *p* < .001, $R_{\text{within}}^{2}$ = .87, $R_{\text{between}}^{2}$ = .07), and negative parent–adolescent relationships were more strongly associated with negative peer relationships (*β* = −.002, *p* < .001, $R_{\text{within}}^{2}$ < .99, $R_{\text{between}}^{2}$ < .01).

##### Adolescent age

Adding adolescent age as a moderator significantly improved the model in two of four analyses: As adolescents got older, supportive parent–adolescent relationships were more strongly associated with supportive peer relationships (*β* = .01, *p =* .003, $R_{\text{within}}^{2}$ = .43, $R_{\text{between}}^{2}$ < .01), and negative parent–adolescent relationships were more strongly associated with negative peer relationships (*β* = .02, *p =* .037, $R_{\text{within}}^{2}$ = .46, $R_{\text{between}}^{2}$ < .01).

##### Additional moderators

Results of the moderator analyses further indicated significant effects for shared informants for some relationship dimensions. Specifically, studies using shared informants (i.e., the same informant reported on the parent–adolescent and peer or romantic relationship) reported larger associations between supportive parent–adolescent and supportive peer relationships compared to studies using non‐shared informants (*β* = .10, *p* = .046, $R_{\text{within}}^{2}$ < .01, $R_{\text{between}}^{2}$ = .08). However, due to little or no variation at one level of the moderator, we could not test the effects of shared informants on the links between negative parent–adolescent relationships and negative or supportive peer relationships (*k* = 1 for non‐shared informant).

Adding population, gender, racial‐ethnic minority, publication status, publication year, or journal impact factor separately as moderators did not significantly improve the model fit for any relationship dimension. Due to little or no variation at some levels of the moderator, we could not test the effects of population on the links between supportive parent–adolescent relationships and negative peer relationships (*k* = 1 for clinical, *k* = 2 for marginalized population) as well as negative parent–adolescent relationships and negative (*k* = 1 for clinical, *k* = 2 for marginalized population) or supportive peer relationships (*k* = 1 for clinical, *k* = 0 for marginalized population). Similarly, we could not test the effects of publication status on the links between supportive or negative parent–adolescent relationships and negative peer relationships (*k* = 1 for unpublished studies).

#### Romantic outcomes

Similar to peer outcomes, we refrain from interpreting all analyses involving controlling parent–adolescent or romantic relationships due to the small number of studies (*k* = 1–4) assessing this relationship dimension (but see Table S4). For all remaining relationship dimensions, parent–adolescent relationship quality was significantly associated with later romantic relationship quality (see Figure 2‐II; Table S3A). More supportive relationships with parents were associated with more supportive (*β* = .11, *p* < .001) and less negative (*β* = −.09, *p* < .001) relationships with romantic partners at the next time point. Similarly, more negative relationships with parents were associated with more negative (*β* = .15, *p* < .001) and less supportive relationships with romantic partners at the next time point (*β* = −.10, *p* < .001).

#### Test of moderators

The analyses involving negative, but not supportive parent–adolescent relationships revealed significant heterogeneity within and across studies (see Table S3A). Levels of heterogeneity were moderate between, but small within studies for the associations between negative parent–adolescent relationships and supportive as well as negative romantic relationships. Therefore, we conducted several analyses to examine whether theoretical moderators (i.e., time lag, adolescent age) and exploratory moderators (i.e., population, shared informant, percentage males, percentage racial‐ethnic minority, publication year, journal impact factor, and journal quartile) significantly explained these differences (see Table S5 for descriptive statistics on all moderators). Contrary to our expectations, none of these moderators significantly improved the model fit for any relationship dimension. Due to little or no variation in the data, we could not test the moderating effects of population and publication status on the associations between negative parent–adolescent relationships and negative (*k* = 2 for clinical, *k* = 3 for marginalized population, and *k* = 2 for unpublished studies) as well as supportive peer relationships (*k* = 1 for clinical, *k* = 2 for marginalized population, and *k* = 1 for unpublished studies).

### Cross‐lagged panel analyses on parent–adolescent and future peer relationships

We used MASEM on all independent effect sizes (*N* = 54) to estimate a cross‐lagged panel model that accounts for concurrent correlations at each time point, stability of each quality measure as well as the reciprocal effect from peer relationship quality to parent–adolescent relationship quality. Missing data on the separate paths were ranged from *n* = 1 to *n* = 8. As too few studies assessed parent–adolescent and romantic relationships simultaneously (*k* = 5), and thus provided stability, concurrent as well as bidirectional correlations, we were unable to estimate a cross‐lagged model for the associations between parent–adolescent and romantic relationships.

#### Main outcomes

The MASEM analyses showed that, controlling for concurrent, stability, and bidirectional correlations, parent–adolescent relationship quality significantly predicted later peer relationship quality for all relationship dimensions (see Figure 3; Table S3B): More supportive parent–adolescent relationships predicted more supportive (*β* = .07, *p* < .001) and less negative (*β* = −.06, *p* = .016) relationships with peers at the next time point. More negative relationships with parents predicted more negative (*β* = .10, *p* = .001) and less supportive (*β* = −.06, *p* = .001) relationships with peers at the next time point. Of four possible reversed effects, only the two effects of peer relationships on negative relationships with parents were significant: More negative relationships with parents were predicted by both more negative (*β* = .06, *p* = .026) and less supportive relationships with peers (*β* = −.06, *p* = .017) at the previous time point. The two bidirectional findings indicated overlapping confidence intervals for the cross‐lagged paths between negative parent–adolescent relationships and negative (CI_par➔peer_ = [.05, .15], CI_peer➔par_ = [.01, .11]) and supportive peer relationships (CI_par➔peer_ = [−.10, −.02], CI_peer➔par_ = [−0.11, −0.01]), which suggests that these paths did not differ from each other. Both parent–adolescent (*β*s = .50–.59, *p*s < .001; see Table S3B) and peer relationship dimensions (*β*s = .37–.43, *p*s < .001) remained quite stable across time, even though studies did not always assess the same peers at *T*_*n*_ and *T*_*n*+1_.

#### Moderator analyses

All analyses revealed significant heterogeneity across studies (see Table S3B). Levels of heterogeneity were substantial for all cross‐lagged paths. Therefore, we added potential theoretical (i.e., adolescent age, time lag) and exploratory moderators (i.e., shared informant, percentage males, percentage racial‐ethnic minority, publication year, journal impact factor, journal quartile) on the structural cross‐lagged parameters to examine whether they explained significant differences between the studies. Most moderators (i.e., adolescent age, time lag, publication year, journal impact factor, journal quartile) significantly improved the model fit for some relationship dimensions (see Table S6). The results of these moderators are depicted in Table S7 (see also Figure 3).

##### Time lag

Adding time lag as a moderator significantly improved the model fit in one of four analyses: However, the time between measurements did not significantly moderate the cross‐lagged associations between supportive parent–adolescent relationships and negative peer relationships (*β*_par➔peer_ = .001, *p* = .451; *β*_peer➔par_ = .01, *p* = .067).

##### Adolescent age

Adding adolescent age as a moderator significantly improved the model fit in three of four analyses: As adolescents got older, the negative association between negative parent–adolescent relationships and supportive peer relationships became stronger (i.e., became more negative; *β* = .02, *p* = .003, $R_{\mathrm{par}\to \text{peer}}^{2}$ = .05). Reversely, the negative association between supportive peer relationships and negative parent–adolescent relationships also became stronger with adolescent age (*β* = .03, *p* = .004, $R_{\text{peer}\to \mathrm{par}}^{2}$ > .99). For the associations between negative parent–adolescent and negative peer relationships as well as between supportive parent–adolescent and negative peer relationships, on the other hand, only the reversed effects were significant: As adolescents got older, the negative association of negative peer relationships with subsequent supportive parent–adolescent relationships became stronger (*β* = .03, *p* = .042, $R_{\text{peer}\to \mathrm{par}}^{2}$ > .99), while the positive association with subsequent negative parent–adolescent relationships *became weaker* (*β* = −.04, *p* = .002, $R_{\text{peer}\to \mathrm{par}}^{2}$ > .99).

##### Additional moderators

Results of the moderator analyses further indicated significant moderation by publication year, journal impact factor, and journal quartile for some relationship dimensions. Compared to older publications, more recent publications reported slightly smaller bidirectional cross‐lagged paths from negative parent–adolescent to supportive peer relationships (*β* = −.004, *p* = .013, $R_{\mathrm{par}\to \text{peer}}^{2}$ = .10) and reversely from supportive peer relationships to negative parent–adolescent relationships (*β* = −.01, *p* = .005, $R_{\text{peer}\to \mathrm{par}}^{2}$ > .90) as well as slightly larger reversed paths from supportive peer to supportive parent–adolescent relationships (*β* = .004, *p* = .019, $R_{\text{peer}\to \mathrm{par}}^{2}$ = .07) as well as from negative peer to negative parent–adolescent relationships (*β* = .01, *p* < .001, $R_{\text{peer}\to \mathrm{par}}^{2}$ = .20).

Adding journal impact factor and journal quartile separately as moderators significantly improved the model fit for some relationship dimensions, but they mainly moderated the reversed paths from peer to parent–adolescent relationships: Publications in journals with higher impact factors reported smaller effects from supportive peer to supportive parent–adolescent relationships (*β* = −.03, *p* = .009, $R_{\text{peer}\to \mathrm{par}}^{2}$ = .05) and from supportive peer to negative parent–adolescent relationships compared to journals with lower impact factors (*β* = −.03, *p* = .009, $R_{\text{peer}\to \mathrm{par}}^{2}$ = .37). Similarly, publications in journals with lower quartile rankings reported larger effects from supportive peer to supportive parent–adolescent relationships (*β* = .04, *p* < .001, $R_{\text{peer}\to \mathrm{par}}^{2}$ = .24), but also reversely from supportive parent–adolescent to supportive peer relationships (*β* = .02, *p* = .029, $R_{\mathrm{par}\to \text{peer}}^{2}$ = .13).

Adding publication status, population, shared informant, gender, or racial‐ethnic minority separately as moderators did not significantly improve the model fit for any relationship dimension. As in the three‐level meta‐regression analyses, due to little or no variation at some levels of the moderator, we could not test the effects of shared informants on the links between negative parent–adolescent relationships and negative or supportive peer relationships and the effects of population on the links between supportive parent–adolescent relationships and negative peer relationships as well as negative parent–adolescent relationships and negative or supportive peer relationships.

### Sensitivity analyses

To disentangle the effects of relationship continuity within (e.g., from supportive parent–adolescent to supportive peer relationships) and across relationship components (e.g., from supportive parent–adolescent to negative peer relationships), we conducted four additional posthoc analyses on the combined samples with inverted scores for the associations across relationship components. Specifically, we analyzed how peer or romantic relationship dimensions moderated the association between parent–adolescent support and peer or romantic relationships (i.e., supportive and negative peer relationships combined) and the association between parent–adolescent negative interaction and peer or romantic relationships. For peer outcomes, supportive parent–adolescent relationships were more strongly associated with supportive than negative peer relationships in the three‐level meta‐regression (*β* = .12, *p* < .001), but not the MASEM analyses (*β* = −.01, *p* = .831). In the MASEM analyses, the reversed effect was significant: Supportive parent–adolescent relationships were more strongly predicted by supportive than negative peer relationships (*β* = .33, *p* < .001). Similarly, negative parent–adolescent relationships were more strongly associated with negative than supportive peer relationships in the three‐level meta‐regression (*β* = .13, *p* < .001), but not the MASEM analyses (*β* = .06, *p* = .060). Again, the reversed effect was significant: Negative parent–adolescent relationships were more strongly predicted by negative than supportive peer relationships (*β* = .29, *p* < .001). For romantic outcomes, supportive parent–adolescent relationships were more strongly associated with supportive than negative romantic relationships (*β* = .03, *p* = .049) and negative parent–adolescent relationships were more strongly associated with negative than supportive romantic relationships (*β* = .10, *p* < .001).

### Publication bias

As many studies in our meta‐analysis did not assess the longitudinal associations between parent–adolescent and peer or romantic relationships as (primary) outcomes, we did not expect publication bias to severely impact our results. Nevertheless, we assessed publication bias and its potential impact on our analyses using two methods. First, visual inspection of the funnel plots and results of Egger's tests suggested that the effects were approximately evenly distributed around the mean in all but one subgroup model, all *p*s > .202 (see Table S8). For the association between negative parent–adolescent and supportive romantic relationships, we detected funnel plot asymmetry (*z* = 2.47, *p* = .013). Second, results from the three‐parameter selection model (*p* value cut point = .05) indicated that the estimated model adjusted for publication bias did not fit the data significantly better than the unadjusted model in all but one of the main analyses, all *p*s > .116. However, the adjusted model seemed to fit better than the unadjusted model for the association between negative parent–adolescent relationships and supportive peer relationships in the three‐level meta‐regression analysis (*r*_adjusted_ = −.01, *p* = .008). The weighted average effect sizes from the other adjusted models were similar to those of the unadjusted models. Due to low variation in effect sizes, we were unable to conduct three‐parameter selection analyses for all but one association involving romantic relationships. However, recent research suggests that publication bias in relatively homogeneous datasets is weak at best (van Aert et al., 2019). Together, these results suggest that some of the subgroup analyses might be subject to publication bias. However, due to the small number of studies, these analyses of publication bias are likely biased and may be false positives (Carter et al., 2019). Combined with our extensive search for unpublished materials and most correlations pertaining to secondary outcomes, publication bias is not likely to severely impact our results.

## DISCUSSION

The present meta‐analysis examined associations between dimensions of parent–adolescent relationships and subsequent peer and romantic relationships in adolescence and emerging adulthood, using two complementary analyses on a range of 10 to 53 studies across analyses. First, we used multilevel analyses to examine how parent–adolescent relationships are associated with future peer and romantic relationships. Second, we used MASEM to examine bidirectional associations between parent–adolescent and future peer relationships, controlling for concurrent associations and over‐time construct stability. Results indicated that supportive and negative parent–adolescent relationships were associated with future supportive and negative peer relationships, above and beyond concurrent associations and construct stability. Also, supportive and negative parent–adolescent relationships were associated with supportive and negative future romantic relationships. For negative parent–adolescent relationships, the transmission from parent–adolescent to peer relationship quality became stronger as adolescents got older. Despite relatively small effect sizes, these findings indicate that parent–adolescent relationship dimensions are significantly related to subsequent peer and romantic relationship dimensions.

### Parent–adolescent relationships as foundation for other social relationships

Consistent with our hypotheses based on social interactional and cognitive theories (e.g., Baldwin, 1992; Bandura, 1977; Furman & Collibee, 2018; Kaufman et al., 2020), we found significant associations across time between parent–adolescent and future peer and romantic relationships within and across relationship dimensions. Specifically, more supportive parent–adolescent relationships were associated with more supportive and less negative peer and romantic relationships. Similarly, more negative parent–adolescent relationships were associated with more negative and less supportive peer and romantic relationships. These findings suggest that core parent–adolescent relationship dimensions are related to subsequent social relationship dimensions. The results from the MASEM analyses further extended these findings, indicating that across time more supportive and more negative parent–adolescent relationships predicted more supportive and more negative future peer relationships, above and beyond concurrent associations and construct stability. These findings support relationship continuity from parent–adolescent to future peer relationships and highlight the importance of both support and negative interaction in the transmission of relationship quality.

While the MASEM results indeed indicated unidirectional pathways from supportive parent–adolescent relationships to peer relationships, they also indicated bidirectional associations between negative parent–adolescent and peer relationships. These bidirectional associations are in line with transactional (Bell, 1968; Sameroff, 2009) and social systems theories (Erel & Burman, 1995), indicating that adolescent experiences with different social partners may spill over to other relationships. The results further suggest that negative interactions might serve a different function than support during adolescence. Adolescence represents a time in which children become more independent and learn to negotiate their own expectations (Collins & Laursen, 2004). Peers become more important and primary relationship partners with whom adolescents practice conflict interactions among equals (Bukowski et al., 2011). These newly practiced and acquired interaction patterns and schemas are thus likely to be generalized to parent–adolescent relationships. Adolescents who are less adept at integrating different objectives and expectations into peer relationships might transfer these negative interaction patterns to the parent–adolescent relationship, which is already susceptible for negative interaction as it realigns toward equality (Branje, 2018). Negative relationship processes increase negative mood, antisocial behaviors and decrease interpersonal competence, through which these negative interactions are likely to spill over to other relationships (Conger et al., 2000; Patterson, 1982), resulting in bidirectional relations between negative interaction patterns in parent–adolescent and peer relationships.

Sensitivity analyses suggested that the transmission of relationship quality from parent–adolescent to peer and romantic relationships was stronger within the same relationship dimensions (e.g., from parent–adolescent support to peer support) than across relationship dimensions (e.g., from parent–adolescent support to peer negative interaction). Similarly, the MASEM findings indicated that the reversed associations from peer relationships to future parent–adolescent relationships were also stronger within than across relationship dimensions. One reason may be that in relationships with parents, adolescents form representations and expectations about social relationships and acquire interaction patterns that they transfer to their relationships with peers and romantic partners (Bandura, 1977). Such representations and interaction patterns are based on specific observed behaviors (e.g., positive or negative relationship behaviors) that form schemata of relationships (Burks & Parke, 1996). In that way, relationship schemata are more easily activated within the same, than across different relationship dimensions (Brewer & Treyens, 1981).

To summarize, the results for both peer and romantic relationships indicate that core dimensions of parent–adolescent relationships continue into other social relationships later in adolescence and emerging adulthood. Even more so, particularly supportive parent–adolescent relationships might provide a foundation for later peer relationships as suggested by dominant theoretical perspectives (e.g., Collins & Sroufe, 1999; Hartup, 1979), while negative parent–adolescent relationships are also predicted by adolescent peer relationships. However, the associations of parent–adolescent relationships with peer and romantic relationships were generally small. These results were in line with previous meta‐analyses on the associations between early parent–child bonds and peer relations (Pallini et al., 2014; Schneider et al., 2001). Relationships with parents during adolescence continue to be associated with later relationships in adolescence and emerging adulthood, and these associations do not seem to become weaker compared to childhood. However, the small effect sizes also suggest that relationship quality with parents does not solely predict later relationship quality with peers and romantic partners. Rather, it is likely that other processes are involved in determining the quality of social relationships (see e.g., Bronfenbrenner & Morris, 2006).

### Moderators in the transmission of relationship quality

#### Effects of time lag

Although we only detected moderating effects of time lag for some relationship dimensions, assessments of parent–adolescent and peer, but not romantic relationships that were further apart in time showed smaller associations. In line with revisionist perspectives (Fraley & Roisman, 2015), increased time between assessments might elicit changes in the parent–adolescent relationship or additional continuous pathways that decrease the association of parent–adolescent relationships with future peer relationships. From a statistical perspective, this finding might reflect serial correlation which specifies that correlations between longitudinal assessments that are closer together in time are stronger than those further apart. Parent–adolescent relationships are thus likely to promptly predict peer relationships.

The associations between parent–adolescent and future romantic relationships did not vary as a function of time intervals, which might suggest stabilizing or enduring effects that are in line with previous empirical findings on the effects of early experiences with parents (Fraley & Roisman, 2015). It may be possible that parent–adolescent relationships provide continuous internal models that are particularly influential for romantic relationships (Sroufe et al., 1990). However, we should note that assessments between parent–adolescent and romantic relationships were generally further apart in time, which resulted in less variation and heterogeneity to detect significant effects between studies. Furthermore, the sample size might have also been too small to detect any potentially significant effects.

While our findings provide preliminary evidence that parent–adolescent relationships transiently affect peer relationships within the same relationship dimension and enduringly affect romantic relationships, we should note that our analyses rendered it difficult to unravel moderating effects of time intervals and adolescent age. It may be possible, for example, that the effects of parent–adolescent relationships in late, compared to early adolescence are more enduring as they realign (Eccles et al., 1993). However, analyses that would assess the effects of time intervals while accounting for adolescent age were too complex to produce reliable, interpretable results. Future studies are needed to find more suitable ways to disentangle potential time and age effects, and thus allow more substantial conclusions about the long‐term effects of parent–adolescent relationships on future peer and romantic relationships. Additionally, parent–adolescent relationships, despite potential changes in their quality, are inherently stable, whereas peer and romantic relationships may dissolve (Laursen & Bukowski, 1997). Most studies, however, did not assess whether relationships with the same peer or romantic partner were assessed across time. Although the perceived quality of peer and romantic relationships remained relatively stable across our analyses, future research might help to unravel potential differences between peer and romantic relationships that remained stable and those that dissolved across time.

#### Effects of adolescent age

Results indicated that adolescent age significantly moderated the links between parent–adolescent relationships and some future peer, but not romantic relationship dimensions. Specifically, as adolescents got older, the longitudinal associations between parent–adolescent and peer relationships within the same relationship dimensions in the three‐level meta‐regressions and negative parent–adolescent and supportive peer relationships in the MASEM analyses became stronger. One reason may be that parent–adolescent relationships become more egalitarian and reciprocal toward late adolescence (Eccles et al., 1993; Hadiwijaya et al., 2017) and thus more similar to peer relationships.

We further detected reversed, but inconsistent effects from peer to parent–adolescent relationships. Specifically, as adolescents got older, supportive and negative parent–adolescent relationships were more weakly predicted by negative peer relationships, but negative parent–adolescent relationships were more strongly predicted by supportive peer relationships. It may be possible that the weaker effect from negative peer to supportive and negative parent–adolescent relationship over time may again reflect the change in peer relationships. Not only do adolescents increasingly turn to peers for support (Blos, 1967; Branje et al., 2021), but these relationships with peers also represent a practice ground for egalitarian relationships (Bukowski et al., 2011). These new egalitarian patterns are likely to spill over to relationships with parents, which themselves become more egalitarian and aligned throughout adolescence. Once parent–adolescent relationships are realigned and established by late adolescence, however, they might become less susceptible to the influence of peer relationships. Alternatively, it may be possible that parents worry less about peer relationships as adolescents get older, whereas they may be more concerned about negative peer interactions at younger ages, resulting in more negative and less supportive parent–adolescent interactions during early to mid‐adolescence (Hadiwijaya et al., 2017). On the other hand, relationships with peers also mature over time in that peers not only mutually and unconditionally support each other, but also learn to accept needs and interests that differ from their own (Selman, 1989). This development of support in peer relationships might positively transfer to parent–adolescent relationships and thus, result in less conflictive, negative interactions. While these findings indicate that there may be some moderating effects of adolescent age, for most relationship dimensions the links between parent–adolescent and future peer relationships did not differ with adolescent age but remained equally strong throughout adolescence and adulthood.

No sample or study characteristics explained differences in how parent–adolescent relationships were associated with future romantic relationships, and only few study characteristics explained differences in how they were associated with future peer relationships. This suggests that the associations between parent–adolescent and future peer and romantic relationships were relatively robust across population, informants, adolescent gender, racial‐ethnic groups, or publication year. For some relationship dimensions, however, we found that studies published in journals with higher impact factors and associated lower quartile rankings (i.e., indicating higher quality journals) reported smaller associations between parent–adolescent and peer relationships compared to journals with lower impact factors and higher quartiles. One reason could be that these journals require studies to implement more rigorous designs and analyses that often result in smaller effect sizes.

### Strengths, limitations, and future directions

This meta‐analysis is the first to synthesize the longitudinal associations between parent–adolescent and future peer and romantic relationships, combining novel meta‐analytic techniques and establishing temporal order. Multilevel meta‐regression allowed us to model the longitudinal associations between parent–adolescent and future peer and romantic relationships, using complete existing evidence from multiple waves of longitudinal studies. Additionally, MASEM extended the multilevel findings and allowed us to additionally model the longitudinal associations between parent–adolescent and future peer relationships in a cross‐lagged panel design, to not only control for concurrent associations and over time stability, but also to investigate potential bidirectional pathways. As such, our findings provide a comprehensive and thorough overview of how core dimensions in parent–adolescent relationships predict the same and other dimensions in later peer and romantic relationships, which helps to build and refine theories on adolescent relationships. While theories generally mark relationships with parents in (early) childhood as most influential for a child's later development, our findings indicate that relationships with parents in adolescence might play an important role as well.

Although this is the most extensive and comprehensive work on the transmission of relationship quality in and beyond adolescence, this meta‐analysis has some limitations. First, as we decided to analyze subgroups of how parent–adolescent relationship dimensions predict the same and other peer and romantic relationship dimensions, some cells in our dataset contained only few effect sizes. While we initially intended to assess how control in parent–adolescent relationships continues into peer and romantic relationships, only two studies assessed control, which did not allow for reliable conclusions. As control is a core relationship dimension, future studies should assess whether and how controlling parent–adolescent relationships continue into future peer and romantic relationships.

Second, while our meta‐analysis focused on the three core relationship dimensions support, negative interaction, and control, the included studies assessed a variety of different constructs that we combined into the three relationship dimensions. It may be possible that some of these aspects exhibit stronger associations than others. Due to the great number of different constructs and combinations in individual studies, we were not able to examine the effects of more specific individual constructs. However, a meta‐analytical review focusing on trust, communication, and alienation as aspects of support, for example, found similarly strong associations across the examined aspects (Gorrese & Ruggieri, 2012).

Third, in some cases, there were too few studies to detect or even test moderation effects. For example, most studies relied solely on adolescents to assess relationship quality. Such reports may be biased as different informants might perceive relationship quality differently. To more reliably and objectively assess longitudinal associations between parent–adolescent and peer or romantic relationships, future longitudinal designs should include multi‐informant ratings of relationship quality. Related to the sample size problem, we were not always able to retrieve all necessary data. From the articles that did not report the required correlations, 28 authors (29%) did not reply to our requests and 22 authors (23%) replied but were not able to reproduce the data. This is a serious problem that may introduce bias not only in conducting meta‐analyses, but in scientific research in general. Open science practices, including data storage and access, help to overcome the problem and should be strongly encouraged, if not required.

Fourth, most studies included samples from White, non‐clinical populations, and Western culture, which might not generalize to samples from other populations or cultures. It could be possible, for example, that the associations between parent–adolescent and peer or romantic relationships are stronger in more collectivistic cultures, in which the family context seems to exert greater influence throughout adolescence and young adulthood (Yoshida & Busby, 2012). Cross‐cultural longitudinal research is needed to provide crucial insights into how the associations between parent–adolescent and other social relationships generalize to other populations and cultures.

Fifth, our meta‐analysis did not allow us to investigate possible differences between mothers and fathers in the association between parent–adolescent and peer or romantic relationships, as such a research question would have required an additional level of dependency in our analyses. Instead, we chose to focus on parent–adolescent relationships more generally. Some studies, however, suggest that relationships with mothers and fathers continue differently into other social relationships (e.g., Möller & Stattin, 2001). Future (meta‐analytic) research might further examine how differences in the relationships with mothers and fathers predict the transmission between parent–adolescent relationship dimensions and later relationships with peers or romantic partners. Relatedly, different types of peer groups, such as classmates or peers, might fulfill different socialization functions (Albarello et al., 2018). Future research might thus investigate how the associations between parent–adolescent and peer or romantic relationships differ among diverse peer groups and romantic partners. Additionally, modes of communication are becoming more diverse with many interactions with peers, romantic partners, and also parents occurring increasingly online. It is possible that online communication, such as social media, offers new opportunities (e.g., meeting platform for peers and romantic partners, sources of support) and challenges (e.g., misperceptions, constant availability, asynchronicity). This may alter the nature and quality of adolescent social relationships (e.g., Nesi et al., 2018). While investigating the effects of different modes of communication on adolescent relationships was beyond the scope of this meta‐analysis, future studies might examine the unique role of new modes of communication and social media in the transmission of relationship quality.

Finally, while our findings established both magnitude and relationship continuity from parent–adolescent to other social relationships, we only examined longitudinal associations that do not permit causal conclusions about the influence of parent–adolescent relationships on peer or romantic relationships. Cross‐lagged models, however, allowed us to establish temporal order between parent–adolescent and peer relationships. As studies rarely assessed parent–adolescent and romantic relationships simultaneously, we cannot rule out that (particularly later) romantic relationships might also be associated with subsequent parent–adolescent relationships. Future longitudinal studies that simultaneously assess parent–adolescent and romantic relationships over time might more clearly indicate whether romantic relationships are indeed preceded by parent–adolescent relationships or whether they also predict them over time.

Relatedly, studies included in this meta‐analysis did not focus on within‐family associations, and thus, dynamic processes that occur within families were not examined. Cross‐lagged models assess rank‐order changes within a group and can thus estimate whether adolescents who have more positive relationships with their parents relative to their peers also develop more positive other social relationships relative to their peers. To assess within‐family dynamics, other designs (e.g., with frequent measurements of discrete parental and adolescent behaviors) or at least other types of models (e.g., dynamic systems approach, Granic, 2005; random‐intercept cross‐lagged panel models, Hamaker et al., 2015) would be required. To infer within‐family dynamics, future empirical research is needed that utilizes such models and designs. The focus of the present study, however, was on the prediction of relative changes, which combine within‐ and between‐family variance.

## CONCLUSION

Adolescence is a critical time in which relationships with peers and romantic partners become increasingly important and relationships with parents become essential prototypes in shaping these new relationships. The results of this meta‐analysis emphasize the importance of positive parent–adolescent relationships for the development of positive peer and romantic relationships in and beyond adolescence. Both supportive and negative parent–adolescent relationships seem to equally predict subsequent peer and romantic relationships. Assisting adolescents and parents in maintaining a mutually supportive relationship may help adolescents to develop positive social relationships that are crucial for their overall well‐being.

## CONFLICT OF INTEREST

None of the authors have any conflicts of interest to disclose.

## Supporting information


Appendix S1

Appendix S2
