# Supplementary material for: The future is present in the past: A meta‐analysis on the longitudinal associations of parent–adolescent relationships with peer and romantic relationships
Source: Child Dev. 2022 Aug 25;94(1):7–27. doi: 10.1111/cdev.13849 (PMC10087754; doi:10.1111/cdev.13849)
Supplement: Supplementary file 1 — Appendix S1 [file CDEV-94-7-s001.docx]

**Supplemental material II - References included in the meta-analysis**

Ajayi, C. A. (2011). *Quarrelsome love: A growth curve analysis of interparental and parent-child relationship factors on romantic relationship conflict in emerging adulthood* [Unpublished doctoral dissertation]. Florida State University.

Allen, J. P., Chango, J., Szwedo, D., & Schad, M. (2014). Long-term sequelae of sub-clinical depressive symptoms in early adolescence. *Development and Psychopathology*, *26*(1), 171–180. https://doi.org/10.1017/S095457941300093X

Allen, J. P., Insabella, G., Porter, M. R., Smith, F. D., Land, D., & Phillips, N. (2006). A social–interactional model of the development of depressive symptoms in adolescence. *Journal of Consulting and Clinical Psychology*, *74*(1), 55–65. https://doi.org/10.1037/0022-006X.74.1.55

Andrews, J. A., Foster, S. L., Capaldi, D., & Hops, H. (2000). Adolescent and family predictors of physical aggression, communication, and satisfaction in young adult couples: A prospective analysis. *Journal of Consulting and Clinical Psychology*, *68*(2), 195–208. https://doi.org/10.1037/0022-006X.68.2.195

Appel, M., Stiglbauer, B., Batinic, B., & Holtz, P. (2014). Internet use and verbal aggression: The moderating role of parents and peers. *Computers in Human Behavior*, *33*, 235–241. https://doi.org/10.1016/j.chb.2014.01.007

Asendorpf, J. B., & van Aken, M. A. G. (2003). Personality-relationship transaction in adolescence: Core versus surface personality characteristics. *Journal of Personality*, *71*(4), 629–666. https://doi.org/10.1111/1467-6494.7104005

Bae, S. M. (2015). The relationships between perceived parenting style, learning motivation, friendship satisfaction, and the addictive use of smartphones with elementary school students of South Korea: Using multivariate latent growth modeling. *School Psychology International*, *36*(5), 513–531. https://doi.org/10.1177/0143034315604017

Baril, H., Julien, D., Chartrand, É., & Dubé, M. (2009). Females’ quality of relationships in adolescence and friendship support in adulthood. *Canadian Journal of Behavioural Science*, *41*(3), 161–168. https://doi.org/10.1037/a0015313

Beach, S. R. H., Lei, K. I. T., Simons, R. L., Barr, A. B., Simons, L. G., Ehrlich, K., … Philibert, R. A. (2017). When inflammation and depression go together: The longitudinal effects of parent-child relationships. *Development and Psychopathology*, *29*, 1969–1986. https://doi.org/10.1017/S0954579417001523

Becht, A. I., Nelemans, S. A., Dijk, M. P. A. Van, Branje, S. J. T., Van Lier, P. A. C., Denissen, J. J. A., & Meeus, W. H. J. (2017). Clear self, better relationships: Adolescents’ self-concept clarity and relationship quality with parents and peers across 5 years. *Child Development*, *88*(6), 1823–1833. https://doi.org/10.1111/cdev.12921

Benner, A. D., Boyle, A. E., & Bakhtiari, F. (2017). Understanding students’ transition to high school: Demographic variation and the role of supportive relationships. *Journal of Youth and Adolescence*, *46*(10), 2129–2142. https://doi.org/10.1007/s10964-017-0716-2

Brendgen, M., Vitaro, F., Tremblay, R. E., & Wanner, B. (2002). Parent and peer effects on delinquency-related violence and dating violence: A test of two mediational models. *Social Development*, *11*(2), 225–244. https://doi.org/10.1111/1467-9507.00196

Brook, J. S., Lee, J. Y., Rubenstone, E., Finch, S. J., Seltzer, N., & Brook, D. W. (2013). Longitudinal determinants of substance use disorders. *Journal of Urban Health: Bulletin of the New York Academy of Medicine*, *90*(6), 1130–1150. https://doi.org/10.1007/s11524-013-9827-6

Burke, T., Sticca, F., & Perren, S. (2017). Everything’s gonna be alright! The longitudinal interplay among social support, peer victimization, and depressive symptoms. *Journal of Youth and Adolescence*, *46*, 1999–2014. https://doi.org/10.1007/s10964-017-0653-0

Burns, A., & Dunlop, R. (1998). Parental divorce, parent-child relations, and early adult relationships: A longitudinal Australian study. *Personal Relationships*, *5*, 393–407. https://doi.org/10.1111/j.1475-6811.1998.tb00178.x

Chen, Z., Liu, R. X., & Kaplan, H. B. (2008). Mediating mechanisms for the intergenerational transmission of constructive parenting. *Journal of Family Issues*, *29*(12), 1574–1599. https://doi.org/10.1177/0192513X08318968

Colarossi, L. G., & Eccles, J. S. (2000). A prospective study of adolescents’ peer support: Gender differences and the influence of parental relationships. *Journal of Youth and Adolescence*, *29*(6), 661–678. https://doi.org/10.1023/A:1026403922442

De Goede, I. H. A., Branje, S. J. T., Delsing, M. J. M. H., & Meeus, W. H. J. (2009). Linkages over time between adolescents’ relationships with parents and friends. *Journal of Youth and Adolescence*, *38*, 1304–1315. https://doi.org/10.1007/s10964-009-9403-2

De Goede, I. H. A., Branje, S., van Duin, J., van der Valk, I. E., & Meeus, W. (2012). Romantic relationship commitment and its linkages with commitment to parents and friends during adolescence. *Social Development*, *21*(3), 425–442. https://doi.org/10.1111/j.1467-9507.2011.00633.x

Drew, H. M. (2017). *Understanding the mental health and well-being of early adolescents in foster care* [Unpublished doctoral dissertation]. University of Sussex.

Dubow, E. F., Huesmann, L. R., Boxer, P., & Smith, C. (2014). Childhood predictors and age 48 outcomes of self-reports and official records of offending. *Criminal Behaviour and Mental Health*, *24*(4), 291–304. https://doi.org/10.1002/cbm.1929

Elmore, G. M., & Huebner, E. S. (2010). Adolescents' satisfaction with school experiences: Relationships with demographics, attachment relationships, and school engagement behavior. *Psychology in the Schools, 47*, 525–537. https://doi.org/10.1002/pits.20488

Fenzel, L. M. (2000). Prospective study of changes in global self-worth and strain during the transition to middle school. *The Journal of Early Adolescence*, *20*(1), 93–116. https://doi.org/10.1177/0272431600020001005

Fosco, G. M., Van Ryzin, M. J., Xia, M., & Feinberg, M. E. (2016). Trajectories of adolescent hostile-aggressive behavior and family climate: Longitudinal implications for young adult romantic relationship competence. *Developmental Psychology*, *52*(7), 1139–1150. https://doi.org/10.1037/dev0000135

Gallagher, M., Prinstein, M. J., Simon, V., & Spirito, A. (2014). Social anxiety symptoms and suicidal ideation in a clinical sample of early adolescents: Examining loneliness and social support as longitudinal mediators. *Journal of Abnormal Child Psychology*, *42*(6), 871–883. https://doi.org/10.1007/s10802-013-9844-7

Gayman, M. D., Turner, R. J., Cislo, A. M., & Eliassen, A. H. (2011). Early adolescent family experiences and perceived social support in young adulthood. *Journal of Early Adolescence*, *31*(6), 880–908. https://doi.org/10.1177/0272431610376247

Giordano, P. C., Cernkovich, S. A., Groat, H. T., Pugh, M. D., & Swinford, S. P. (1998). The quality of adolescent friendships: Long term effects? *Journal of Health and Social Behavior*, *39*(1), 55–71. https://doi.org/10.2307/2676389

Goodnight, J. A., Bates, J. E., Holtzworth-Munroe, A., Pettit, G. S., Ballard, R. H., Iskander, J. M., … Lansford, J. E. (2017). Dispositional, demographic, and social predictors of trajectories of intimate partner aggression in early adulthood. *Journal of Consulting and Clinical Psychology*, *85*(10), 950–965. https://doi.org/10.1037/ccp0000226

Gore, S., & Aseltine, R. H. (1995). Protective processes in adolescence: Matching stressors with social resources. *American Journal of Community Psychology*, *23*(3), 301–327. https://doi.org/10.1007/BF02506947

Guan, S.-S. A., & Fuligni, A. J. (2016). Changes in parent, sibling, and peer support during the transition to young adulthood. *Journal of Research on Adolescence*, *26*(2), 286–299. https://doi.org/10.1111/jora.12191

Guay, F., Denault, A., & Renauld, S. (2017). School attachment and relatedness with parents, friends and teachers as predictors of students’ intrinsic and identified regulation. *Contemporary Educational Psychology*, *51*, 416–428. https://doi.org/10.1016/j.cedpsych.2017.10.001

Guay, F., Marsh, H. W., Senécal, C., & Dowson, M. (2008). Representations of relatedness with parents and friends and autonomous academic motivation during the late adolescence-early adulthood period: reciprocal or unidirectional effects? *The British Journal of Educational Psychology*, *78*, 621–637. https://doi.org/10.1348/000709908X280971

Guinn, T. D. (2012). *Emerging adult friendship: A consequence of family communication and catalyst for well-being* [Unpublished doctoral dissertation]. University of Texas

Hazel, N., Oppenheimer, C., Technow, J., Young, J., & Hankin, B. (2014). Parent relationship quality buffers against the effect on depressive symptoms from middle childhood to adolescence. *Developmental Psychology*, *50*(8), 2115–2123. https://doi.org/10.1037/a0037192.Parent

Herd, T., Li, M., Maciejewski, D., Lee, J., Deater-Deckard, K., King-Casas, B., & Kim-Spoon, J. (2018). Inhibitory control mediates the association between perceived stress and secure relationship quality. *Frontiers in Psychology*, *9*, 217. https://doi.org/10.3389/fpsyg.2018.00217

Holt, L. J., Mattanah, J. F., & Long, M. W. (2018). Change in parental and peer relationship quality during emerging adulthood: Implications for academic, social, and emotional functioning. *Journal of Social and Personal Relationships*, *35*(5), 743–769. https://doi.org/10.1177/0265407517697856

Ivanova, K. O. (2012). *From parents to partners: The impact of family on romantic relationships in adolescence and emerging adulthood* [Unpublished doctoral dissertation]. University of Groningen.

Katz, S. J., Hammen, C. L., & Brennan, P. A. (2013). Maternal depression and the intergenerational transmission of relational impairment. *Journal of Family Psychology*, *27*(1), 86–95. https://doi.org/10.1037/a0031411

Kaufman-Parks, A. M., DeMaris, A., Giordano, P. C., Manning, W. D., & Longmore, M. A. (2017). Parents and partners: Moderating and mediating influences on intimate partner violence across adolescence and young adulthood. *Journal of Social and Personal Relationships*, *34*(8), 1295–1323. https://doi.org/10.1177/0265407516676639

Kaufman-Parks, A. M., DeMaris, A., Giordano, P. C., Manning, W. D., & Longmore, M. A. (2018). Familial effects on intimate partner violence perpetration across adolescence and young adulthood. *Journal of Family Issues*, *39*(7), 1933–1961. https://doi.org/10.1177/0192513X17734586

Kim, H., & Pears, K. (2009). Emotion dysregulation in the intergenerational transmission of romantic relationship conflict. *Journal of Family Psychology*, *23*(4), 585–595. https://doi.org/10.1037/a0015935.Emotion

Kochendorfer, L. B., & Kerns, K. A. (2017). Perceptions of parent-child attachment relationships and friendship qualities: Predictors of romantic relationship involvement and quality in adolescence. *Journal of Youth and Adolescence*, *46*(5), 1009–1021. https://doi.org/10.1007/s10964-017-0645-0

Kogan, S. M., Lei, M.-K., Grange, C. R., Simons, R. L., Brody, G. H., Gibbons, F. X., & Chen, Y. (2013). The contribution of community and family contexts to African American young adults’ romantic relationship health: A prospective analysis. *Journal of Youth and Adolescence*, *42*(6), 878–890. https://doi.org/10.1007/s10964-013-9935-3

Kretschmer, T., Sentse, M., Meeus, W., Verhulst, F. C., Veenstra, R., & Oldehinkel, A. J. (2016). Configurations of adolescents’ peer experiences: Associations with parent–child relationship quality and parental problem behavior. *Journal of Research on Adolescence*, *26*(3), 474–491. https://doi.org/10.1111/jora.12206

Kretschmer, T., Vollebergh, W., & Oldehinkel, A. J. (2017). Parent–child positivity and romantic relationships in emerging adulthood: Congruence, compensation, and the role of social skills. *International Journal of Behavioral Development*, *41*(2), 198–210. https://doi.org/10.1177/0165025415612228

Laird, R. D., Bridges, B. J., & Marsee, M. A. (2013). Secrets from friends and parents: Longitudinal links with depression and antisocial behavior. *Journal of Adolescence*, *36*(4), 685–693. https://doi.org/10.1016/j.adolescence.2013.05.001

Laursen, B., Furman, W., & Mooney, K. S. (2006). Predicting interpersonal competence and self-worth from adolescent relationships and relationship networks: Variable-centered and person-centered perspectives. *Merrill-Palmer Quarterly*, *52*(3), 572–600. https://doi.org/10.1353/mpq.2006.0030

Lee, S.-A. (2018). Parental divorce, relationships with fathers and mothers, and children’s romantic relationships in young adulthood. *Journal of Adult Development*, *25*(2), 121–134. https://doi.org/10.1007/s10804-017-9279-4

Letcher, P., Smart, D., Sanson, A., & Toumbourou, J. W. (2009). Psychosocial precursors and correlates of differing internalizing trajectories from 3 to 15 years. *Social Development*, *18*(3), 618–646. https://doi.org/10.1111/j.1467-9507.2008.00500.x

Linder, J. R., & Collins, W. A. (2005). Parent and peer predictors of physical aggression and conflict management in romantic relationships in early adulthood. *Journal of Family Psychology*, *19*(2), 252–262. https://doi.org/10.1037/0893-3200.19.2.252

Lohman, B. J., Neppl, T. K., Senia, J. M., & Schofield, T. J. (2013). Understanding adolescent and family influences on intimate partner psychological violence during emerging adulthood and adulthood. *Journal of Youth and Adolescence*, *42*(4), 500–517. https://doi.org/10.1007/s10964-013-9923-7

Luijpers, E. T. H. (2000). *Intention to explore, social bonds and delinquent behaviour of Dutch adolescents* [Unpublished doctoral dissertation]. Utrecht University.

Luyckx, K., Missotten, L., Goossens, E., & Moons, P. (2012). Individual and contextual determinants of quality of life in adolescents with congenital heart disease. *Journal of Adolescent Health*, *51*(2), 122–128. https://doi.org/10.1016/j.jadohealth.2011.11.007

Mak, H. W., Fosco, G. M., & Feinberg, M. E. (2018). The role of family for youth friendships: Examining a social anxiety mechanism. *Journal of Youth and Adolescence*, *47*(2), 306–320. https://doi.org/10.1007/s10964-017-0738-9

Makin-Byrd, K., Bierman, K. L., & Conduct Problems Prevention Research Group. (2013). Individual and family predictors of the perpetration of dating violence and victimization inlate adolescence. *Journal of Youth and Adolescence*, *42*(4), 536–550. https://doi.org/10.1007/s10964-012-9810-7

Martin, M. J., Davies, P. T., & Cummings, E. M. (2017). Distinguishing attachment and affiliation in early adolescents’ narrative descriptions of their best friendship. *Journal of Research on Adolescence*, *27*(3), 644–660. https://doi.org/10.1111/jora.12305

Meeus, W. H. J., Branje, S. J. T., van der Valk, I., & de Wied, M. (2007). Relationships with intimate partner, best friend, and parents in adolescence and early adulthood: A study of the saliency of the intimate partnership. *International Journal of Behavioral Development*, *31*(6), 569–580. https://doi.org/10.1177/0165025407080584

Meeus, W. H. J., Branje, S., & Overbeek, G. J. (2004). Parents and partners in crime: A six-year longitudinal study on changes in supportive relationships and delinquency in adolescence and young adulthood. *Journal of Child Psychology and Psychiatry*, *45*(7), 1288–1298. https://doi.org/10.1111/j.1469-7610.2004.00312.x

Mize, J. L., & Kliewer, W. (2018). Domain-specific daily hassles, anxiety, and delinquent behaviors among low-income, urban youth. *Journal of Applied Developmental Psychology*, *53*, 31–39. https://doi.org/10.1016/j.appdev.2017.09.003

Morris, A. M., Mrug, S., & Windle, M. (2015). From family violence to dating violence: Testing a dual pathway model. *Journal of Youth and Adolescence*, *44*(9), 1819–1835. https://doi.org/10.1007/s10964-015-0328-7

Mumford, E. A., Liu, W., & Taylor, B. G. (2016). Parenting profiles and adolescent dating relationship abuse: Attitudes and experiences. *Journal of Youth and Adolescence*, *45*(5), 959–972. https://doi.org/10.1007/s10964-016-0448-8

Murphy, D. A., Marelich, W. D., Lanza, H. I., & Herbeck, D. M. (2012). Effects of maternal HIV on children's psychosocial adjustment with peers and with their mother. *Vulnerable children and youth studie*s*, 7*(4), 357–370. https://doi.org/10.1080/17450128.2012.708461

Musliner, K. L., & Singer, J. B. (2014). Emotional support and adult depression in survivors of childhood sexual abuse. *Child Abuse and Neglect*, *38*(8), 1331–1340. https://doi.org/10.1016/j.chiabu.2014.01.016

Nummer, G., & Seiffge-Krenke, I. (2001). Können Unterschiede in Stresswahrnehmung und -bewältigung Geschlechtsunterschiede in der depressiven Symptombelastung bei Jungendlichen erklären? [Can differences in recognition of and coping with stress explain gender differences in depressive symptoms]. *Zeitschrift Für Kinder- Und Jugendpsychiatrie Und Psychotherapie*, *29*(2), 89–97. https://doi.org/10.1024//1422-4917.29.2.89

Pinquart, M., & Pfeiffer, J. P. (2013). Perceived social support in adolescents with and without visual impairment. *Research in Developmental Disabilities*, *34*(11), 4125–4133. https://doi.org/10.1016/j.ridd.2013.08.004

Puckett, M. B. (2010). *Rejection sensitivity and interpersonal relationship difficulties: Depression, loneliness, and self-esteem as mediating factors* [Unpublished doctoral dissertation]. University of Connecticut.

Rauer, A. J., Pettit, G. S., Lansford, J. E., Bates, J. E., & Dodge, K. A. (2013). Romantic relationship patterns in young adulthood and their developmental antecedents. *Developmental Psychology*, *49*(11), 2159–2171. https://doi.org/10.1037/a0031845

Rice, K. G., & Mulkeen, P. (1995). Relationships with parents and peers: A longitudinal study of adolescent intimacy. *Journal of Adolescent Research*, *10*(3), 338–357. https://doi.org/10.1177/0743554895103003

Rodríguez, S. A., Perez-Brena, N. J., Updegraff, K. A., & Umaña-Taylor, A. J. (2014). Emotional closeness in mexican-origin adolescents’ relationships with mothers, fathers, and same-sex friends. *Journal of Youth and Adolescence*, *43*(12), 1953–1968. https://doi.org/10.1007/s10964-013-0004-8

Rosario, M., Salzinger, S., Feldman, R. S., & Ng-Mak, D. S. (2008). Intervening processes between youths’ exposure to community violence and internalizing symptoms over time: The roles of social support and coping. *American Journal of Community Psychology*, *41*(1–2), 43–62. https://doi.org/10.1007/s10464-007-9147-7

Scharf, M., & Mayseless, O. (2008). Late adolescent girls’ relationships with parents and romantic partner: The distinct role of mothers and fathers. *Journal of Adolescence*, *31*, 837–855. https://doi.org/10.1016/j.adolescence.2008.06.012

Schmidt, C. & Seiffge-Krenke, I. (1996). Freundschaftsbeziehungen und familiäre Beziehungen aus der Sicht chronisch kranker und gesunder Jugendlicher: Unterschiede in den Beziehungsqualitäten und Veränderungen über die Zeit [Perceptions of friendships and family relations in chronically III and healthy adolescents: Quality of relationships and change over time]. *Psychologie in Erziehung und Unterricht, 43,* 155-168.

Seiffge-Krenke, I. (2003). Testing theories of romantic development from adolescence to young adulthood: Evidence of a developmental sequence. *International Journal of Behavioral Development*, *27*(6), 519–531. https://doi.org/10.1080/01650250344000145

Seiffge-Krenke, I., & Persike, M. (2017). Gendered pathways to young adult symptomatology: The impact of managing relationship stress during adolescence. *International Journal of Behavioral Development*, *41*(1), 52–63. https://doi.org/10.1177/0165025416646485

Slominski, L., Sameroff, A., Rosenblum, K., & Kasser, T. (2011). Longitudinal pathways between maternal mental health in infancy and offspring romantic relationships in adulthood: A 30-year prospective study. *Social Development*, *20*(4), 762–782. https://doi.org/10.1111/j.1467-9507.2011.00610.x

Song, J., Bong, M., Lee, K., & Kim, S. (2015). Longitudinal investigation into the role of perceived social support in adolescents’ academic motivation and achievement. *Journal of Educational Psychology*, *107*(3), 821–841. https://doi.org/10.1037/edu0000016

Starks, T. J., Newcomb, M. E., & Mustanski, B. (2015). A longitudinal study of interpersonal relationships among lesbian, gay, and bisexual adolescents and young adults: Mediational pathways from attachment to romantic relationship quality. *Archives of Sexual Behavior*, *44*(7), 1821–1831. https://doi.org/10.1007/s10508-015-0492-6

Stocker, C. M., & Richmond, M. K. (2007). Longitudinal associations between hostility in adolescents’ family relationships and friendships and hostility in their romantic relationships. *Journal of Family Psychology*, *21*(3), 490–497. https://doi.org/10.1037/0893-3200.21.3.490

Surjadi, F. F., Lorenz, F. O., Conger, R. D., & Wickrama, K. A. S. (2013). Harsh, inconsistent parental discipline and romantic relationships: Mediating processes of behavioral problems and ambivalence. *Journal of Family Psychology*, *27*(5), 762–772. https://doi.org/10.1037/a0034114

Sweeten, G., Larson, M., & Piquero, A. R. (2016). Predictors of emotional and physical dating violence in a sample of serious juvenile offenders. *Criminal Behaviour and Mental Health*, *26*, 263–277. https://doi.org/10.1002/cbm

Tillinger, M. (2013). *The influence of friends and family on well-being for children and adolescents with developmental disabilities* [Unpublished doctoral dissertation]. Boston College.

Tyrell, F. A., Wheeler, L. A., Gonzales, N. A., Dumka, L., & Millsap, R. (2014). Family influences on Mexican American adolescents’ romantic relationships: Moderation by gender and culture. *Journal of Research on Adolescence*, *26*(1), 142–158. https://doi.org/10.1111/jora.12177

Umemura, T., & Šerek, J. (2016). Different developmental pathways from parental warmth to adolescents’ trust in peers and politicians: Mediating roles of adolescent-parent attachment and belief in a just world. *Social Justice Research*, *29*, 186–205. https://doi.org/10.1007/s11211-016-0258-x

Van Zalk, N., & Van Zalk, M. (2015). The importance of perceived care and connectedness with friends and parents for adolescent social anxiety. *Journal of Personality*, *83*(3), 346–360. https://doi.org/10.1111/jopy.12108

Walper, S., & Wendt, E.-V. (2015). Adolescents’ relationships with mother and father and their links to the quality of romantic relationships: A classification approach. *European Journal of Developmental Psychology*, *12*(5), 516–532. https://doi.org/10.1080/17405629.2015.1065727

Wray-Lake, L., Syvertsen, A. K., & Flanagan, C. A. (2016). Developmental change in social responsibility during adolescence: An ecological perspective. *Developmental Psychology*, *52*(1), 130–142. https://doi.org/10.1037/dev0000067.supp

Yeung Thompson, R. S., & Leadbeater, B. J. (2013). Peer victimization and internalizing symptoms from adolescence into young adulthood: Building strength through emotional support. *Journal of Research on Adolescence*, *23*(2), 290–303. https://doi.org/10.1111/j.1532-7795.2012.00827.x.Peer

Zhang, S., Baams, L., van de Bongardt, D., & Dubas, J. S. (2018). Intra- and inter-individual differences in adolescent depressive mood: The role of relationships with parents and friends. *Journal of Abnormal Child Psychology*, *46*, 811–824. https://doi.org/10.1007/s10802-017-0321-6
