# Supplementary material for: The future is present in the past: A meta‐analysis on the longitudinal associations of parent–adolescent relationships with peer and romantic relationships
Source: Child Dev. 2022 Aug 25;94(1):7–27. doi: 10.1111/cdev.13849 (PMC10087754; doi:10.1111/cdev.13849)
Supplement: Supplementary file 2 — Appendix S2 [file CDEV-94-7-s002.docx]

**Supplemental material**

# Search for unpublished materials

Based on best practices and recommendations by Cochrane and the Campbell Collaboration (see Bonato, 2018), we conducted a comprehensive search of unpublished materials that spanned 15 databases on dissertations (ProQuest Dissertations, Networked Digital Library of Theses and Dissertations, DART Europe), conference proceedings (OAIster, Scopus, Web of Science), policy documents (Policy Commons, Overton), preregistrations (OSF preregistrations, SocArXiv, PsyArXiv) and other grey literature (Grey literature in the Netherlands, Nederlandse Bibliografie Online, OpenGrey, GreyNet). We used the same search strategy and search strings that we used for our search for published studies (see exact search strings below on pp. 2-7 in the supplemental material). In total, we identified 1,381 unpublished documents, which we screened for title and abstract as well as full text (see Figure S1). Of 184 documents that we screened for full text, 170 were dissertations, four were reports, seven were conference papers, and three were presentations. In cases in which we could not retrieve the full text, we contacted the first author. Out of 30 contacted authors, only three authors replied to our request and one of those authors provided the requested text. Based on the full-text screening, we included 67 studies, of which we excluded a number of documents that had already been published (*n* = 27) or used data that overlapped with studies included in our meta-analysis (*n* = 23). For included studies that did not report the required effect sizes (*n* = 11), we contacted the authors using the same strategy as for published studies. Of all 10 authors contacted, three provided the requested effect sizes, but two of those studies had to be excluded because the relationship constructs combined several relationship dimensions. Seven authors did not reply to our request or could not provide the requested correlations. Hence, nine studies were excluded, resulting in a final inclusion of seven unpublished dissertations.

# Search strings for all databases

# The exact search strings used for this meta-analytic review are provided below.

***EBSCOHOST (ERIC, PsycArticles, PsycInfo)***

SU ( adolescen* OR teen* OR youth OR youngst* OR student* OR “emerging adult*” OR “early adult*” OR “young adult*” ) AND SU ( Famil* OR parent* OR mother* OR father* OR maternal OR paternal ) AND SU ( friend* OR peer* OR romantic* OR intimate* OR marital OR marriage OR couple* ) AND SU ( Relation* OR dating OR warmth OR bond OR affecti* OR attachment OR intimacy OR nurturance OR sensitivity OR support OR aggression OR conflict OR discord OR hostility OR discipline OR abuse OR victimization OR violence ) AND SU ( Longitudinal* OR prospective OR intergenerational OR transmission OR spillover OR “spill*_over” ) Journal or Document: Journal Articles (EJ);

***SCOPUS***

( TITLE-ABS-KEY ( adolescen* OR teen* OR youth "emerging adult*" OR "early adult*" OR "young adult*" ) AND TITLE-ABS-KEY ( famil* OR parent* OR mother* OR father* OR maternal OR paternal ) AND TITLE-ABS-KEY ( friend* OR peer* OR romantic* OR intimate* OR marital OR marriage OR couple* ) AND TITLE-ABS-KEY ( relation* OR dating OR warmth OR bond OR affecti* OR attachment OR intimacy OR nurturance OR sensitivity OR support OR aggression OR conflict OR discord OR hostility OR discipline OR abuse OR victimization OR violence ) AND TITLE-ABS-KEY ( longitudinal* OR prospective OR intergenerational OR transmission OR spillover OR "spill*_over" ) ) AND aggression OR conflict OR discord OR hostility OR discipline OR abuse OR victimization OR violence ) AND ( EXCLUDE ( SUBJAREA , "MEDI" ) ) AND ( LIMIT-TO ( DOCTYPE , "ar" ) OR LIMIT-TO ( DOCTYPE , "ip" ) )

***Web of Science***

TOPIC:(adolescen* OR teen* OR youth OR youngst* OR student* OR “emerging adult*” OR “early adult*” OR “young adult*”) AND TOPIC: (Famil* OR parent* OR mother* OR father* OR maternal OR paternal) AND TOPIC:(friend* OR peer* OR romantic* OR intimate* OR marital OR marriage OR couple*) AND TOPIC: (Relation* OR dating OR warmth OR bond OR affecti* OR attachment OR intimacy OR nurturance OR sensitivity OR support OR aggression OR conflict OR discord OR hostility OR discipline OR abuse OR victimization OR violence) AND TOPIC: (Longitudinal* OR prospective OR intergenerational OR transmission OR spillover OR “spill*_over”) Refined by: DOCUMENT TYPES: ( ARTICLE ) Timespan: All years. Indexes: SSCI.

***Networked Digital Library of Theses and Dissertations***

description:(adolescen* OR teen* OR youth OR youngst* OR student* OR “emerging adult*” OR “early adult*” OR “young adult*”) AND (famil* OR parent* OR mother* OR father* OR maternal OR paternal) AND (friend* OR peer* OR romantic* OR intimate* OR marital OR marriage OR couple*) AND (relation* OR dating OR warmth OR bond OR affecti* OR attachment OR intimacy OR nurturance OR sensitivity OR support OR aggression OR conflict OR discord OR hostility OR discipline OR abuse OR victimization OR violence) AND (longitudinal* OR prospective OR intergenerational OR transmission OR spillover OR “spill*_over”)

***OAIster***

kw:(adolescen* OR teen* OR youth OR youngst* OR student* OR “emerging adult*” OR “early adult*” OR “young adult*”) AND (famil* OR parent* OR mother* OR father* OR maternal OR paternal) AND (friend* OR peer* OR romantic* OR intimate* OR marital OR marriage OR couple*) AND (relation* OR dating OR warmth OR bond OR affecti* OR attachment OR intimacy OR nurturance OR sensitivity OR support OR aggression OR conflict OR discord OR hostility OR discipline OR abuse OR victimization OR violence) AND (longitudinal* OR prospective OR intergenerational OR transmission OR spillover OR “spill*_over”)' > '1900..2018' *Limited to* Libraries Worldwide

***OpenGrey***

abstract: (adolescen* OR teen* OR youth OR youngst* OR student* OR “emerging adult*” OR “early adult*” OR “young adult*”) AND abstract: (famil* OR parent* OR mother* OR father* OR maternal OR paternal) AND abstract: (friend* OR peer* OR romantic* OR intimate* OR marital OR marriage OR couple*) AND abstract: (relation* OR dating OR warmth OR bond OR affecti* OR attachment OR intimacy OR nurturance OR sensitivity OR support OR aggression OR conflict OR discord OR hostility OR discipline OR abuse OR victimization OR violence) AND abstract: (longitudinal* OR prospective OR intergenerational OR transmission OR spillover OR “spill*_over”)

(abstract: adolescen* OR teen* OR youth OR youngst* OR student* OR “emerging adult*” OR “early adult*” OR “young adult*”) AND (abstract: famil* OR parent* OR mother* OR father* OR maternal OR paternal) AND (abstract: friend* OR peer* OR romantic* OR intimate* OR marital OR marriage OR couple*) AND (abstract: relation* OR dating OR warmth OR bond OR affecti* OR attachment OR intimacy OR nurturance OR sensitivity OR support OR aggression OR conflict OR discord OR hostility OR discipline OR abuse OR victimization OR violence) AND (abstract: longitudinal* OR prospective OR intergenerational OR transmission OR spillover OR “spill*_over”)

***Policy Commons***

(adolescen* OR teen* OR youth OR youngst* OR student* OR “emerging adult*” OR “early adult*” OR “young adult*”) AND (famil* OR parent* OR mother* OR father* OR maternal OR paternal) AND (friend* OR peer* OR romantic* OR intimate* OR marital OR marriage OR couple*) AND (relation* OR dating OR warmth OR bond OR affecti* OR attachment OR intimacy OR nurturance OR sensitivity OR support OR aggression OR conflict OR discord OR hostility OR discipline OR abuse OR victimization OR violence) AND (longitudinal* OR prospective OR intergenerational OR transmission OR spillover OR “spill*_over”)

***Overton***

abstract: (adolescen* OR teen* OR youth OR youngst* OR student* OR “emerging adult*” OR “early adult*” OR “young adult*”) AND (famil* OR parent* OR mother* OR father* OR maternal OR paternal) AND (friend* OR peer* OR romantic* OR intimate* OR marital OR marriage OR couple*) AND (relation* OR dating OR warmth OR bond OR affecti* OR attachment OR intimacy OR nurturance OR sensitivity OR support OR aggression OR conflict OR discord OR hostility OR discipline OR abuse OR victimization OR violence) AND (longitudinal* OR prospective OR intergenerational OR transmission OR spillover OR “spill*_over”)

***DART Europe***

Keywords = (adolescen* OR teen* OR youth OR youngst* OR student* OR “emerging adult*” OR “early adult*” OR “young adult*”) AND (famil* OR parent* OR mother* OR father* OR maternal OR paternal) AND (friend* OR peer* OR romantic* OR intimate* OR marital OR marriage OR couple*) AND (relation* OR dating OR warmth OR bond OR affecti* OR attachment OR intimacy OR nurturance OR sensitivity OR support OR aggression OR conflict OR discord OR hostility OR discipline OR abuse OR victimization OR violence) + refined by (longitudinal* OR prospective OR intergenerational OR transmission OR spillover OR “spill*_over”)

(adolescen* OR teen* OR youth OR youngst* OR student*) AND (famil* OR parent* OR mother* OR father* OR maternal OR paternal) AND (friend* OR peer* OR romantic* OR intimate* OR marital OR marriage OR couple*) AND (relation* OR dating OR warmth OR bond OR affecti* OR attachment OR intimacy OR nurturance OR sensitivity OR support OR aggression OR conflict OR discord OR hostility OR discipline OR abuse OR victimization OR violence) AND (longitudinal* OR prospective OR intergenerational OR transmission OR spillover)

***ProQuest***

ab(adolescen* OR teen* OR youth OR youngst* OR student* OR “emerging adult*” OR “early adult*” OR “young adult*”) AND ab(famil* OR parent* OR mother* OR father* OR maternal OR paternal) AND ab(friend* OR peer* OR romantic* OR intimate* OR marital OR marriage OR couple*) AND ab(relation* OR dating OR warmth OR bond OR affecti* OR attachment OR intimacy OR nurturance OR sensitivity OR support OR aggression OR conflict OR discord OR hostility OR discipline OR abuse OR victimization OR violence) AND ab(longitudinal* OR prospective OR intergenerational OR transmission OR spillover OR “spill*_over”)

***GLIN***

(adolescen* OR teen* OR youth OR youngst* OR student* OR “emerging adult*” OR “early adult*” OR “young adult*”) AND (famil* OR parent* OR mother* OR father* OR maternal OR paternal) AND (friend* OR peer* OR romantic* OR intimate* OR marital OR marriage OR couple*) AND (relation* OR dating OR warmth OR bond OR affecti* OR attachment OR intimacy OR nurturance OR sensitivity OR support OR aggression OR conflict OR discord OR hostility OR discipline OR abuse OR victimization OR violence) AND (longitudinal* OR prospective OR intergenerational OR transmission OR spillover OR “spill*_over”)

***Nederlandse Bibliografie Online***

(adolescen* OR teen* OR youth OR youngst* OR student* OR “emerging adult*” OR “early adult*” OR “young adult*”) AND (famil* OR parent* OR mother* OR father* OR maternal OR paternal) AND (friend* OR peer* OR romantic* OR intimate* OR marital OR marriage OR couple*) AND (relation* OR dating OR warmth OR bond OR affecti* OR attachment OR intimacy OR nurturance OR sensitivity OR support OR aggression OR conflict OR discord OR hostility OR discipline OR abuse OR victimization OR violence) AND (longitudinal* OR prospective OR intergenerational OR transmission OR spillover OR “spill*_over”)

***OSF preregistrations***

(adolescen* OR teen* OR youth OR youngst* OR student* OR “emerging adult*” OR “early adult*” OR “young adult*”) AND (famil* OR parent* OR mother* OR father* OR maternal OR paternal) AND (friend* OR peer* OR romantic* OR intimate* OR marital OR marriage OR couple*) AND (relation* OR dating OR warmth OR bond OR affecti* OR attachment OR intimacy OR nurturance OR sensitivity OR support OR aggression OR conflict OR discord OR hostility OR discipline OR abuse OR victimization OR violence) AND (longitudinal* OR prospective OR intergenerational OR transmission OR spillover OR “spill*_over”)

***PsyArXiv / SocArXiv***

(adolescen* OR teen* OR youth OR youngst* OR student* OR “emerging adult*” OR “early adult*” OR “young adult*”) AND (famil* OR parent* OR mother* OR father* OR maternal OR paternal) AND (friend* OR peer* OR romantic* OR intimate* OR marital OR marriage OR couple*) AND (relation* OR dating OR warmth OR bond OR affecti* OR attachment OR intimacy OR nurturance OR sensitivity OR support OR aggression OR conflict OR discord OR hostility OR discipline OR abuse OR victimization OR violence) AND (longitudinal* OR prospective OR intergenerational OR transmission OR spillover OR “spill*_over”)

# Information about the coding process

*Coding procedure*

Coding decisions were based on a coding protocol that was developed prior to the coding process by the first and second author, in consultation with three experts in the field of adolescent family and peer relationships. The protocol included all coding categories and information about how studies should be scored based on these categories. Before the final coding, the protocol was piloted across 7 studies by the first and second author. Based on this piloting phase, we discussed diverging ratings with all experts and adjusted the protocol accordingly. Disagreements in the piloting and coding phase were presented to the experts and discussed among the experts and raters until consensus was reached among all experts. That mainly concerned the inclusion and categorization of relationship constructs.

To operationalize the relationship dimensions (i.e., support, negative interaction, and control), we developed a list of keywords on often-assessed relationship constructs that corresponded to these broad dimensions (see below). This list was developed in agreement with experts and based on the theoretical framing of the three relationship dimensions. For each study, we consulted the description of each included construct and the used instrument to allocate it to one of the three dimensions. We only included constructs that corresponded to relationship quality, for example if the assessed behaviors or constructs characterized relationships with parents, peers, or partners, or if they were directed from one relationship partner towards the other. Study reports that did not focus on specific relationships, but on social competence or behavior in general (e.g., “makes friends easily”, “plays with other children”) were excluded.

Similarly, study reports that used a very broad construct (e.g., relationship quality) that did not allow us to disentangle different dimensions or a construct that combined several aspects of different dimensions, such as autonomy (dimension of control) and relatedness (dimension of support), were excluded if these constructs could not be separated. We also detected constructs that we could not allocate to one of the three relationship dimensions, such as ‘information management’ (for studies focusing on communication aspects, information exchange between relationship partners, including disclosure or secrecy). Due to the specific focus of our meta-analysis and limits regarding manuscript length, these additional dimensions were not included in the present study.

Additionally, most studies (87.5%) did not provide information about whether the assessment of peer relationships referred to the same or different relationships at T1 and T2. Of those that did, most studies indicated including a mixture of both stable and unstable peer relationships (57.1%), and only a small proportion of studies assessed the same peer relationships across time (14.3%). As assessments of relationships mostly focus on perceptions of relationships, however, which are likely to remain similar even across different peers, we still recorded stability coefficients for these different relationships.

*List with keywords that correspond to specific relationship dimensions*

| Support | Control | Negative interaction |
| --- | --- | --- |
| Commitment | Dominance | Negative interaction |
| Satisfaction | Control | Aggression |
| Trust | Autonomy | Hostility |
| Identification | Power | Rejection |
| Connectedness | Dating restriction | Violence (including physical and psychological victimization or perpetration) |
| Attraction | Overprotection | Antagonism |
| Warmth |  | Criticism |
| Intimacy | Directing of friendships | Threat/abuse |
| Sensitivity | Psychological control | Punishment/discipline |
| Relatedness | Jealousy | Hassles |
| Companionship |  | Problems |
| Alienation (reverse-coded) |  | Derogation |
| Affection |  | (Di)stress |
| Emotional support |  | Coercion |
| Closeness |  | Disagreements |
| Nurture/care |  |  |
| Acceptance |  |  |
| Affiliation |  |  |

*Interrater reliabilities for all main outcomes*

| Category | 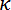 | ICC |
| --- | --- | --- |
| *Publication* |  |  |
| Journal IS | 1 |  |
| Journal IF |  | 1 |
| *Study procedures* |  |  |
| Country | 1 |  |
| Recruitment location | 1 |  |
| Recruitment strategy | .993 |  |
| Attrition across waves | 1 |  |
| # Waves family |  | 1 |
| # Waves friends |  | 1 |
| # Waves romantic |  | 1 |
| Time lag between measures |  | 1 |
| *Sample* |  |  |
| N adolescents fam T1 |  | .992 |
| N adolescents rom/peer T1 |  | .999 |
| M_age_ adolescent T1 |  | 1 |
| M_age_ adolescent T2 |  | .928 |
| Population | .990 |  |
| % Adolescent boys |  | .979 |
| % Racial-ethnic minority |  | .988 |
| Living with both parents |  | .995 |
| Living with single parents |  | .995 |
| Degree of friendship | 1 |  |
| Type of romantic sexuality | 1 |  |
| Same peer/romantic partner across waves | .80 |  |
| *Measures* |  |  |
| Relationship dimension parent-adolescent | 1 |  |
| Relationship dimension peer/romantic | 1 |  |
| Construct name parent-adolescent | .992 |  |
| Construct name peer/rom | .991 |  |
| Family member | .979 |  |
| Informant parent-adolescent | 1 |  |
| Informant peer/romantic | 1 |  |
| Instrument parent-adolescent | 1 |  |
| Instrument peer/rom | 1 |  |
| IRR parent-adolescent | 1 |  |
| IRR peer/romantic | 1 |  |
| *Effect sizes* |  |  |
| Effect size |  | .997 |
| Sample size N |  | 1 |

# Table S1A *Sample Descriptives for Peer Relationships*

| Study (Year) | *N* | # waves family | # waves peer | Time lag (in mo) | Age T_n_ | % boys | % rac-eth minority | Region | Study cohort | Population | Publication status |
| --- | --- | --- | --- | --- | --- | --- | --- | --- | --- | --- | --- |
| Allen et al. (2006) | 143 | 2 | 2 | 12.00 | 13.34 | 48.25 | 37.00 | North America | study specific | community | published |
| Appel et al. (2014) | 363 | 2 | 2 | 6.00 | 12.84 | 45.70 |  | Western Europe | EARSandEYES | community | published |
| Asendorpf & van Aken (2003) | 167 | 2 | 2 | 36.00 | 12.00 | 51.74 |  | Western Europe | Munich Longitudinal Study on the Genesis of Individual Competencies | community | published |
| Bae, 2015 | 2264 | 1 | 3 | 12.00 |  | 52.20 |  | Asia | Korea Youth Panel Survey | community | published |
| Baril et al. (2009) | 29 | 1 | 1 | 84.00 | 14.63 | 0.00 | 0.00 | North America | study specific | community | published |
| Becht et al. (2017) | 450 | 5 | 5 | 12.00 | 13.03 | 57.00 | 5.20 | Western Europe | Research on Adolescent Development and Relationships (RADAR) | community | published |
| Benner et al. (2017) | 138 | 2 | 2 | 12.00 | 14.38 | 50.00 | 98.00 | North America | Schools, Peers, and Adolescent Development Project | racial-ethnic minority | published |
| Burke et al. (2017) | 814 | 4 | 4 | 6.00 | 13.20 | 51.00 |  | Western Europe | net-TEEN | community | published |
| Colarossi & Eccles (2000) | 198 | 1 | 1 | 48.00 | 11.50 | 48.10 | 10.00 | North America | study specific | community | published |

| Study (Year) | *N* | # waves family | # waves peer | Time lag (in mo) | Age T_n_ | % boys | % rac-eth minority | Region | Study cohort | Population | Publication status |
| --- | --- | --- | --- | --- | --- | --- | --- | --- | --- | --- | --- |
| De Goede et al. (2009, sample A) | 923 | 5 | 5 | 12.00 | 12.40 | 54.10 | 15.50 | Western Europe | Conflict and Management Of Relationships (CONAMORE) | community | published |
| De Goede et al. (2009, sample B) | 390 | 5 | 5 | 12.00 | 16.70 | 43.30 | 15.50 | Western Europe | Conflict and Management Of Relationships (CONAMORE) | community | published |
| Drew (2017) | 555 | 2 | 2 | 6.00 | 12.00 | 46.49 |  | Western Europe | study specific | community | unpublished |
| Elmore & Huebner (2010) | 398 | 2 | 2 | 12.00 |  | 39.60 | 56.80 | North America | study specific | community | published |
| Fenzel (2000) | 118 | 2 | 2 | 3.00 | 10.80 | 43.97 | 10.00 | North America | study specific | community | published |
| Gallagher et al. (2014) | 144 | 2 | 2 | 9.00 | 13.51 | 28.00 | 25.00 | North America | study specific | clinical | published |
| Gayman et al. (2011) | 1267 | 1 | 1 | 96.00 | 12.00 | 74.00 | 71.00 | North America | study specific | community | published |
| Giordano et al. (1998) | 620 | 2 | 2 | 120.00 | 15.30 | 44.00 | 49.00 | North America | study specific | marginalized | published |
| Gore & Aseltine (1995) | 1036 | 2 | 2 | 12.00 | 15.50 | 43.40 | 0.00 | North America | study specific | community | published |
| Guan & Fuligni (2016) | 478 | 3 | 3 | 24.00 | 17.81 | 48.90 | 80.50 | North America | study specific | community | published |
| Guay et al. (2008) | 380 | 3 | 3 | 12.00 | 17.70 | 28.30 |  | North America | study specific | community | published |
| Guay et al. (2017) | 639 | 2 | 2 | 12.00 |  | 42.00 |  | North America | study specific | marginalized | published |
| Guinn (2012) | 272 | 1 | 2 | 3.00 | 18.12 | 35.66 | 51.70 | North America | study specific | community | unpublished |

| Study (Year) | *N* | # waves family | # waves peer | Time lag (in mo) | Age T_n_ | % boys | % rac-eth minority | Region | Study cohort | Population | Publication status |
| --- | --- | --- | --- | --- | --- | --- | --- | --- | --- | --- | --- |
| Hazel et al. (2014, sample A) | 243 | 1 | 5 | 3.00 | 11.00 | 44.45 | 32.35 | North America | study specific | marginalized | published |
| Hazel et al. (2014, sample B) | 223 | 1 | 5 | 3.00 | 14.60 | 44.45 | 32.35 | North America | study specific | minority | published |
| Herd et al. (2018) | 167 | 2 | 2 | 24.00 | 14.13 | 53.00 | 20.00 | North America | study specific | community | published |
| Holt et al. (2018, sample A) | 153 | 2 | 2 | 48.00 | 18.60 | 36.00 | 27.00 | North America | study specific | community | published |
| Holt et al. (2018, sample B) | 138 | 2 | 2 | 48.00 | 17.67 | 28.00 | 32.00 | North America | study specific | community | published |
| Kretschmer et al. (2016) | 1806 | 1 | 1 | 60.00 | 11.00 | 49.00 | 10.00 | Western Europe | Tracking Adolescents’ Individual Lives Survey (TRAILS) | community | published |
| Laird et al. (2013) | 163 | 2 | 2 | 12.00 | 12.40 | 49.50 | 60.70 | North America | study specific | community | published |
| Laursen et al. (2006) | 188 | 2 | 2 | 24.00 | 15.30 | 50.00 | 30.50 | North America | study specific | community | published |
| Letcher et al. (2009) | 1141 | 6 | 2 | 24.00 | 13.00 | 51.90 |  | Oceania | Australian Temperament Project (ATP) | community | published |
| Luijpers (2000, sample A) | 387 | 2 | 2 | 36.00 | 13.00 | 46.86 |  | Western Europe | Wendingen in de Levensloop | community | unpublished |
| Luijpers (2000, sample B) | 444 | 2 | 2 | 36.00 | 16.00 | 46.86 |  | Western Europe | Wendingen in de Levensloop | community | unpublished |

| Study (Year) | *N* | # waves family | # waves peer | Time lag (in mo) | Age T_n_ | % boys | % rac-eth minority | Region | Study cohort | Population | Publication status |
| --- | --- | --- | --- | --- | --- | --- | --- | --- | --- | --- | --- |
| Luyckx et al. (2012) | 429 | 2 | 2 | 9.00 | 15.75 | 53.40 |  | Western Europe | Information technology Devices and Education programme for Transitioning Adolescents with Congenital Heart disease  (i-DETACH) | community | published |
| Mak et al. (2018) | 567 | 3 | 3 | 18.00 | 11.27 | 47.70 | 10.00 | North America | Promoting School-Community-University Partnerships to Enhance Resilience (PROSPER) | community | published |
| Martin et al. (2017) | 278 | 1 | 3 | 12.00 | 13.00 | 50.00 | 73.00 | North America | study specific | community | published |
| Meeus et al. (2007) | 350 | 2 | 2 | 72.00 | 17.59 | 42.00 | 1.00 | Western Europe | Utrecht Study of Adolescent Development (USAD) | community | published |
| Mize & Kliewer (2018, sample A) | 167 | 2 | 2 | 12.00 | 10.78 | 48.40 | 91.40 | North America | Project COPE | marginalized | published |
| Mize & Kliewer (2018, sample B) | 146 | 2 | 2 | 12.00 | 13.68 | 43.70 | 92.00 | North America | Project COPE | marginalized | published |
| Murphy et al. (2012) | 107 | 11 | 11 | 6.00 | 13.00 | 52.00 | 95.00 | North America | Parents and Children Coping Together (PACT) | minority | published |

| Study (Year) | *N* | # waves family | # waves peer | Time lag (in mo) | Age T_n_ | % boys | % rac-eth minority | Region | Study cohort | Population | Publication status |
| --- | --- | --- | --- | --- | --- | --- | --- | --- | --- | --- | --- |
| Musliner & Singer (2014) | 15409 | 2 | 1 | 168.00 | 14.90 | 52.80 | 28.30 | North America | National Longitudinal Study of Adolescent Health (Add Health) | community | published |
| Nummer & Seiffge-Krenke (2001) | 210 | 4 | 4 | 12.00 | 13.90 | 46.80 |  | Western Europe | study specific | community | published |
| Pinquart & Pfeiffer (2013, sample A) | 134 | 3 | 3 | 12.00 | 15.64 | 44.00 |  | Western Europe | Marburg Study of Vision Loss | clinical | published |
| Pinquart & Pfeiffer (2013, sample B) | 422 | 3 | 3 | 12.00 | 14.19 | 48.00 |  | Western Europe | Marburg Study of Vision Loss | community | published |
| Puckett (2010) | 371 | 2 | 2 | 12.00 | 13.95 | 42.10 | 21.00 | North America | study specific | community | unpublished |
| Rauer et al. (2013) | 511 | 1 | 1 | 48.00 | 12.00 | 51.00 | 16.00 | North America | Child Development Project | community | published |
| Rice & Mulkeen (1995) | 109 | 3 | 3 | 48.00 | 13.00 | 47.71 | 0.00 | North America | study specific | community | published |
| Rodríguez et al. (2014) | 246 | 3 | 3 | 24.00 | 12.51 | 49.00 | 100.00 | North America | study specific | minority | published |
| Rosario et al. (2008) | 613 | 3 | 3 | 12.00 | 11.80 | 50.22 | 97.00 | North America | study specific | marginalized | published |
| Schmidt & Seiffge-Krenke (1996, sample A) | 53 | 3 | 3 | 24.00 | 13.90 |  |  | Western Europe | Chronisch kranke Jugendliche und ihre Familien | community | published |

| Study (Year) | *N* | # waves family | # waves peer | Time lag (in mo) | Age T_n_ | % boys | % rac-eth minority | Region | Study cohort | Population | Publication status |
| --- | --- | --- | --- | --- | --- | --- | --- | --- | --- | --- | --- |
| Schmidt & Seiffge-Krenke (1996, sample B) | 36 | 3 | 3 | 24.00 | 13.90 |  |  | Western Europe | Chronisch kranke Jugendliche und ihre Familien | clinical | published |
| Seiffge-Krenke (2003) | 103 | 3 | 3 | 24.00 | 13.00 | 40.80 |  | Western Europe | study specific | community | published |
| Seiffge-Krenke & Persike (2017) | 145 | 2 | 2 | 48.00 | 13.60 | 44.80 | 7.00 | Western Europe | study specific | community | published |
| Song et al. (2015) | 6089 | 2 | 2 | 12.00 |  | 51.80 |  | Asia | Korean Educational Longitudinal Study | community | published |
| Starks et al. (2015) | 186 | 3 | 3 | 6.00 | 18.80 | 53.40 | 86.30 | North America | study specific | community | published |
| Tillinger (2013) | 93 | 1 | 1 | 96.00 | 10.00 | 47.30 | 10.90 | North America | Early Intervention Collaborative Study (EICS) | clinical | unpublished |
| Umemura & Šerek (2016) | 409 | 1 | 1 | 48.00 | 13.00 | 50.00 |  | Eastern Europe | European Longitudinal Study of Pregnancy and Childhood | community | published |
| Wray-Lake et al. (2016) | 4150 | 3 | 3 | 12.00 | 13.70 | 44.60 | 28.20 | North America | Social Responsibility and Prevention Project | community | published |
| Yeung Thompson & Leadbeater (2013, sample A) | 361 | 3 | 3 | 24.00 | 13.50 |  | 15.00 | North America | Victoria Healthy Youth Survey | community | published |
| Yeung Thompson & Leadbeater (2013, sample B) | 278 | 3 | 3 | 24.00 | 17.00 |  | 15.00 | North America | Victoria Healthy Youth Survey | community | published |
| Zhang et al. (2018) | 945 | 4 | 4 | 6.00 | 13.95 | 53.00 | 20.80 | Western Europe | Studies on Trajectories of Adolescent Relationships and Sexuality (STARS) | community | published |

*Note. N* = sample size, time lag = time between measurements (in months), age T_n_ = adolescent age at first included measurement (in years), rac-eth = racial-ethnic, community = mostly racial-ethnic majority, non-clinical sample, clinical = clinical sample (including mental or physical disabilities), minority = racial-ethnic minority sample (including refugees), marginalized = economically marginalized, such as low income sample, empty cells = missing data.

# Table S1B *Sample Descriptives for Romantic Relationships*

| Study (Year) | *N* | # waves family | # waves romantic | Time lag (in mo) | Age T_n_ | % boys | % rac-eth minority | Region | Study cohort | Population | Publication status |
| --- | --- | --- | --- | --- | --- | --- | --- | --- | --- | --- | --- |
| Ajayi (2011) | 3346 | 1 | 4 | 60.00 | 15.21 | 49.10 | 48.00 | North America | National Longitudinal Survey of Youth (NLSY97) | community | unpublished |
| Allen et al. (2014) | 184 | 2 | 1 | 84.00 | 15.30 | 46.70 | 42.00 | North America | study specific | community | published |
| Andrews et al. (2000) | 135 | 1 | 1 | 72.00 | 16.70 | 42.00 | 7.00 | North America | study specific | community | published |
| Beach et al. (2017) | 380 | 1 | 2 | 126.00 | 10.50 |  | 100.00 | North America | Family and Community Health Study (FACHS) | minority | published |
| Brendgen et al. (2002) | 336 | 1 | 1 | 54.00 | 12.00 | 100.00 |  | North America | study specific | community | published |
| Brook et al. (2013) | 816 | 1 | 1 | 168.00 | 19.20 | 40.00 | 100.00 | North America | study specific | minority | published |
| Burns & Dunlop (1998) | 72 | 3 | 1 | 120.00 | 14.70 | 52.60 |  | Oceania | study specific | community | published |
| Chen et al. (2008) | 1560 | 1 | 1 | 156.00 | 12.80 | 37.00 |  | North America | study specific | community | published |
| De Goede et al. (2012, sample A) | 218 | 5 | 1 | 48.00 | 12.40 | 39.00 | 21.00 | Western Europe | Conflict and Management Of Relationships (CONAMORE) | community | published |
|  |  |  |  |  |  |  |  |  |  |  |  |
| Study (Year) | *N* | # waves family | # waves romantic | Time lag (in mo) | Age T_n_ | % boys | % rac-eth minority | Region | Study cohort | Population | Publication status |
| De Goede et al. (2012, sample B) | 185 | 5 | 1 | 48.00 | 16.70 | 30.80 | 22.00 | Western Europe | Conflict and Management Of Relationships (CONAMORE) | community | published |
| Fosco et al. (2016) | 106 | 1 | 2 | 6.00 | 16.38 | 39.40 | 43.20 | North America | study specific | community | published |
| Giordano et al. (1998) | 620 | 1 | 1 | 120 | 15.30 | 44.00 | 49.00 |  | study specific | marginalized | published |
| Goodnight et al. (2017) | 240 | 1 | 6 | 24.00 | 16.00 | 44.00 | 19.00 | North America | Child Development Project | community | published |
| Ivanova (2012) | 131 | 3 | 1 | 120.00 | 15.00 | 56.80 |  | Western Europe | Solna study | community | unpublished |
| Katz et al. (2013) | 182 | 1 | 1 | 60.00 | 15.00 | 36.26 | 4.00 | North America | Mater University Study of Pregnancy | clinical | published |
| Kaufman-Parks et al. (2017) | 950 | 5 | 5 | 12.00 | 15.22 | 46.60 | 34.10 | North America | Toledo Adolescent Relationships Study (TARS) | community | published |
| Kaufman-Parks et al. (2018) | 950 | 5 | 5 | 12.00 | 15.22 | 46.60 | 34.10 | North America | Toledo Adolescent Relationships Study (TARS) | community | published |
| Kim & Pears (2009) | 190 | 1 | 1 | 156.00 | 15.50 | 100.00 | 10.00 | North America | Oregon Youth Study (OYS) | community | published |
| Kochendorfer & Kerns (2017) | 192 | 1 | 2 | 24.00 | 10.00 | 63.00 | 28.10 | North America | National Institute of Child Health and Human Development Study of Early Child Care and Youth Development (NICHD SECCYD) | community | published |

| Study (Year) | *N* | # waves family | # waves romantic | Time lag (in mo) | Age T_n_ | % boys | % rac-eth minority | Region | Study cohort | Population | Publication status |
| --- | --- | --- | --- | --- | --- | --- | --- | --- | --- | --- | --- |
| Kogan et al. (2013) | 285 | 2 | 1 | 134.40 | 10.40 | 40.60 | 100.00 | North America | Family and Community Health Study (FACHS) | minority | published |
| Kretschmer et al. (2017) | 811 | 1 | 1 | 132.00 | 11.10 | 49.00 | 10.00 | Western Europe | Tracking Adolescents’ Individual Lives Survey (TRAILS) | community | published |
| Laursen et al. (2006) | 167 | 2 | 2 | 24.00 | 15.30 | 50.00 | 30.50 | North America | study specific | community | published |
| Lee (2018, sample A) | 520 | 1 | 1 | 72.00 | 18.00 | 40.20 | 6.00 | North America | Michigan Study of Adolescent Life Transitions | community | published |
| Lee (2018, sample B) | 154 | 1 | 1 | 72.00 | 18.00 | 30.50 | 6.00 | North America | Michigan Study of Adolescent Life Transitions | community | published |
| Linder & Collins (2005) | 81 | 1 | 2 | 96.00 | 13.00 | 47.93 | 32.20 | North America | Minnesota Longitudinal Study of Parents and Children | marginalized | published |
| Lohman et al. (2013) | 392 | 1 | 2 | 78.00 | 14.50 | 48.00 | 0.00 | North America | Family Transitions Project (FTP) | community | published |
| Makin-Byrd et al. (2013) | 401 | 1 | 1 | 60.00 |  | 57.00 | 41.60 | North America | Fast Track Multisite Investigation | community | published |

| Study (Year) | *N* | # waves family | # waves romantic | Time lag (in mo) | Age T_n_ | % boys | % rac-eth minority | Region | Study cohort | Population | Publication status |
| --- | --- | --- | --- | --- | --- | --- | --- | --- | --- | --- | --- |
| Meeus et al. (2004, sample A) | 362 | 3 | 3 | 36.00 | 15.80 | 42.00 | 1.00 | Western Europe | Utrecht Study of Adolescent Development (USAD) | community | published |
| Meeus et al. (2004, sample B) | 280 | 3 | 3 | 36.00 | 22.50 | 42.00 | 1.00 | Western Europe | Utrecht Study of Adolescent Development (USAD) | community | published |
| Morris et al. (2015) | 461 | 1 | 1 | 72.00 | 11.79 | 49.00 | 81.00 | Western Europe | Birmingham Youth Violence Study (BYVS) | community | published |
| Mumford et al. (2016) | 1117 | 1 | 2 | 12.00 | 15.30 | 52.80 | 39.60 | North America | Survey on Teen Relationships and Intimate Violence (STRiV) | community | published |
| Puckett (2010) | 371 | 2 | 2 | 12.00 | 13.95 | 42.10 | 21.00 | North America | study specific | community | unpublished |
| Scharf & Mayseless (2008) | 49 | 1 | 2 | 18.00 | 17.50 | 0.00 | 0.00 | Eastern Europe | study specific | community | published |
| Seiffge-Krenke et al. (2003) | 44 | 3 | 1 | 96.00 | 13.00 | 40.80 | 2.00 | Western Europe | study specific | community | published |
| Slominski et al. (2011, sample A) | 52 | 2 | 1 | 204.00 | 13.00 | 45.00 | 32.00 | North America | study specific | community | published |
| Slominski et al. (2011, sample B) | 41 | 2 | 1 | 204.00 | 13.00 | 45.00 | 32.00 | North America | study specific | clinical | published |
| Starks et al. (2015) | 150 | 3 | 3 | 12.00 | 18.80 | 53.40 | 86.30 | North America | study specific | community | published |
| Stocker & Richmond (2007) | 110 | 1 | 1 | 36.00 | 15.06 | 51.00 | 20.00 | North America | study specific | community | published |

| Study (Year) | *N* | # waves family | # waves romantic | Time lag (in mo) | Age T_n_ | % boys | % rac-eth minority | Region | Study cohort | Population | Publication status |
| --- | --- | --- | --- | --- | --- | --- | --- | --- | --- | --- | --- |
| Surjadi et al. (2013) | 288 | 1 | 1 | 156.00 | 15.00 | 43.80 | 0.00 | North America | Family Transitions Project (FTP) | community | published |
| Sweeten et al. (2016) | 617 | 1 | 1 | 48.00 | 17.60 | 100.00 | 80.30 | North America | Pathways to Desistance Study | community | published |
| Tyrell et al. (2014) | 142 | 1 | 1 | 24.00 | 12.29 | 46.00 | 100.00 | North America | study specific | minority | published |
| Walper & Wendt (2015) | 684 | 1 | 1 | 12.00 | 18.60 | 42.40 |  | Western Europe | Panel Analysis of Intimate Relationships and Family Dynamics (PAIRFAM) | community | published |

*Note. N* = sample size, time lag = time between measurements (in months), age T_n_ = adolescent age at first included measurement (in years), rac-eth = racial-ethnic, community = mostly racial-ethnic majority, non-clinical sample, clinical = clinical sample (including mental or physical disabilities), minority = racial-ethnic minority sample (including refugees), marginalized = economically marginalized, such as low income sample, empty cells = missing data.

# Table S2. *Interpretative Overview of All Significant Main and Moderation Outcomes*

|  |  | *Three-level random effects models – peer outcomes* | | | | | | | | | | | |
| --- | --- | --- | --- | --- | --- | --- | --- | --- | --- | --- | --- | --- | --- |
|  |  | *Meta-analytic effect size* | | | *Moderators* | | | | | | | | |
| **Parent T_n_** | **Peer T_n+1_** |  | ***r*** | ***p*** | ***time*** | ***age*** | ***population*** | ***shared*** | ***boys*** | ***rac-eth*** | ***year*** | ***J_IF_*** | ***J_Q_*** |
| support | support |  | + | sig | − | + |  | + |  |  |  |  |  |
|  | negative |  | − | sig |  |  |  | N/A |  |  |  |  |  |
| negative | support |  | − | sig |  |  |  |  |  |  |  |  |  |
|  | negative |  | + | sig | − | + |  | N/A |  |  |  |  |  |
|  |  |  |  |  |  |  |  |  |  |  |  |  |  |
|  |  | *Three-level random effects models – romantic outcomes* | | | | | | | | | |  |  |
| support | support |  | + | sig | No heterogeneity in effect sizes  No heterogeneity in effect sizes | | | | | | | | |
|  | negative |  | − | sig |  |  |  |  |  |  |  |  |  |
| negative | support |  | − | sig |  |  |  |  |  |  |  |  |  |
|  | negative |  | + | sig |  |  |  |  |  |  |  |  |  |
|  |  |  |  |  |  |  |  |  |  |  |  |  |  |
|  |  | *Random effects structural equation models – peer outcomes* | | | | | | | | | |  |  |
| support | support | CL_par>peer_  CL_peer>par_ | +  + | sig | − |  |  |  |  |  | + | − | + |
|  | negative | CL_par>peer_  CL_peer>par_ | −  − | sig |  | + |  | N/A |  |  |  |  |  |
| negative | support | CL_par>peer_  CL_peer>par_ | −  − | sig  sig |  | + + |  |  |  |  | + + | − |  |
|  | negative | CL_par>peer_  CL_peer>par_ | +  + | sig  sig |  | − |  | N/A |  |  | −  − |  |  |

*Note.* Time = time between measurements (in months), age = adolescent age, shared = shared informant (i.e., the same informant reported on parent-adolescent and peer or romantic relationship), year = publication year, boys = percentage of boys, rac-eth = racial-ethnic minority, population = community, marginalized, or clinical sample, J_IF_ = journal impact factor, J_Q_ = journal quartile, sig = significant, CL_par>peer_ *=* cross-lagged path from parent T_n_ to peer T_n+1_, CL_peer>par_ *=* cross-lagged path from peer to parent, empty cells = not significant, N/A = too few studies available.

# Table S3A. *Summary of All Main Three-level Meta-regression Analyses*

|  |  | *Three-level random effects models – peer outcomes* | | | | | | | | |
| --- | --- | --- | --- | --- | --- | --- | --- | --- | --- | --- |
|  |  |  |  | *Meta-analytic effect size* | | | *Heterogeneity estimates* | | | |
| Parent T_n_ | Peer T_n+1_ | *k* | *n* ES | β | *p* | 95% CI | *Q* | *p_q_* | *I_w_* | *I_b_* |
| support | support | 53 | 174 | .18 | <.001 | [.15, .20] | 902.16 | <.001 | 9.6% | 68.9% |
| support | negative | 11 | 53 | −.12 | <.001 | [−.16, −.07] | 115.23 | <.001 | <0.01% | 73.4% |
| negative | support | 13 | 51 | −.07 | .002 | [−.12, −.03] | 188.67 | <.001 | 2.7% | 74.2% |
| negative | negative | 11 | 52 | .18 | <.001 | [.12, .24] | 163.32 | <.001 | 7.4% | 73.7% |
|  |  |  |  |  |  |  |  |  |  |  |
|  |  | *Three-level random effects models – romantic outcomes* | | | | | | | | |
|  |  |  |  | *Meta-analytic effect size* | | | *Heterogeneity estimates* | | | |
| Parent T_n_ | Rom T_n+1_ | *k* | *n* ES | β | *p* | 95% CI | *Q* | *p_q_* | *I_w_* | *I_b_* |
| support | support | 18 | 43 | .11 | <.001 | [.09, .13] | 44.57 | .364 | <0.01% | <0.01% |
| support | negative | 10 | 24 | −.09^[[1]](#footnote-1)^ | <.001 | [−.14, −.07] | 19.59 | .666 | <0.01% | <0.01% |
| negative | support | 10 | 25 | −.10 | <.001 | [−.15, −.05] | 49.04 | .002 | 18.8% | 50.1% |
| negative | negative | 17 | 32 | .15 | <.001 | [.11, .19] | 206.48 | <.001 | 14.5% | 66.0% |

*Note. k* = number of independent samples, *n* ES = number of effect sizes, CI = lower and upper limits of confidence interval, *Q* = heterogeneity test, *p_q_* = *p* value of heterogeneity statistic, *I_w_* = percentage of within-study heterogeneity, *I_b_* = percentage of between-study heterogeneity.

# Table S3B. *Summary of All Main MASEM Analyses*

|  |  | *Random effects structural equation models – peer outcomes* | | | | | | | | | | | |  | |
| --- | --- | --- | --- | --- | --- | --- | --- | --- | --- | --- | --- | --- | --- | --- | --- |
|  |  | *Meta-analytic effect size* | | | | | | | | | | | | *Heterogeneity* | |
|  |  | CL par>peer | *p* | CL  peer>par | *p* | Stab  parent | *p* | Stab  peer | *p* | CS  T_n_ | *p* | CS T_n+1_ | *p* | *Q* | *p* |
| support on  support (*k*=53) | | .07 | <.001 | .01 | .324 | .55 | <.001 | .41 | <.001 | .23 | <.001 | .16 | <.001 | 2966.58 | <.001 |
| support on  negative (*k*=11) | | −.06 | .016 | −.02 | .333 | .59 | <.001 | .39 | <.001 | −.15 | <.001 | −.02 | .403 | 199.3 | <.001 |
| negative on  support (*k*=13) | | −.06 | .001 | −.06 | .017 | .54 | <.001 | .43 | <.001 | −.02 | .572 | −.04 | .322 | 323.32 | <.001 |
| negative on negative (*k*=11) | | .10 | .001 | .06 | .026 | .50 | <.001 | .37 | <.001 | .25 | <.001 | .18 | <.001 | 399.40 | <.001 |

*Note.* CL par>peer = cross-lagged path from parent T_n_ to peer T_n+1_, CL peer>par = cross-lagged path from peer T_n_ to parent T_n+1_, Stab parent = stability path from parent T_n_ to parent T_n+1_*,* Stab peer = stability path from peer T_n_ to peer T_n+1_, CS T_n_ = concurrent association between parent T_n_ and peer T_n_, CS T_n+1_ = concurrent association between parent T_n+1_ and peer T_n+1_.

# Table S4. *Summary of Main Three-level Meta-Regression Analyses involving Control*

|  |  | *Three-level random effects models – peer outcomes* | | | | | | | | |
| --- | --- | --- | --- | --- | --- | --- | --- | --- | --- | --- |
|  |  |  |  | *Meta-analytic effect size* | | | *Heterogeneity estimates* | | | |
| Parent T_n_ | Peer T_n+1_ | *k* | *n ES* | β | *p* | 95% CI | *Q* | *p_q_* | *I_w_* | *I_b_* |
| control | control | 2 | 20 | .240 | <.001 | [.209, .270] | 43.35 | .001 | 48.6% | 5.7% |
| control | support | 1 | 21 | .036 | <.001 | [.020, .053] | 13.17 | .870 | - | - |
| control | negative | 2 | 20 | .101 | <.001 | [.076, .127] | 32.28 | .029 | 32.7% | 3.2% |
| support | control | 2 | 20 | −.055 | .002 | [−.090, −.019] | 17.26 | .573 | <.01% | 23.5% |
| negative | control | 2 | 20 | .105 | <.001 | [.065, .145] | 26.41 | .119 | 3.9% | 28.8% |
|  |  |  |  |  |  |  |  |  |  |  |
|  |  | *Three-level random effects models – romantic outcomes* | | | | | | | | |
|  |  |  |  | *Meta-analytic effect size* | | | *Heterogeneity estimates* | | | |
| Parent T_n_ | Rom T_n+1_ | *k* | *n* ES | β | *p* | 95% CI | *Q* | *p_q_* | *I_w_* | *I_b_* |
| *control* | *control* | *1* | *1* | *−.075* | *.049* | *[−.150, −.000]* | *-* | *-* | *-* | *-* |
| *control* | *support* | *4* | *5* | *−.030* | *.210* | *[−.076, .017]* | *6.99* | *.136* | *<0.01%* | *<0.01%* |
| *control* | *negative* | *4* | *5* | *.047* | *NA* | *NA* | *6.74* | *.150* | *<0.01%* | *<0.01%* |
| *support* | *control* | *1* | *1* | *.145* | *<.001* | *[.072, .218]* | *-* | *-* | *-* | *-* |
| negative | control | 2 | 11 | .015 | .816 | [−.109, −.139] | 43.22 | <.001 | 18.3% | 69.4% |

*Note. k* = number of independent samples, *n* ES = number of independent effect sizes*,* 95% CI = lower and upper limits of 95% confidence interval, *Q* = heterogeneity test, *p_q_* = *p* value of heterogeneity statistic, *I_w_* = percentage of within-study heterogeneity, *I_b_* = percentage of between-study heterogeneity. Fit attempts for italicized estimates resulted in errors.

# Table S5. *Descriptive Statistics for Moderator Variables included in Subgroup Analyses*

|  | *Peer outcomes* | | | | *Romantic outcomes* | | | |
| --- | --- | --- | --- | --- | --- | --- | --- | --- |
|  | Frequency (%) or *M* (SD) | | | | Frequency (%) or *M* (SD) | | | |
| Moderator | support T_n_ 🡪 support T_n+1_ | support T_n_ 🡪 negative T_n+1_ | Negative T_n_ 🡪 support T_n+1_ | Negative T_n_ 🡪 negative T_n+1_ | support T_n_ 🡪 support T_n+1_ | support T_n_ 🡪 negative T_n+1_ | Negative T_n_ 🡪 support T_n+1_ | Negative T_n_ 🡪 negative T_n+1_ |
| Time lag (in months) | 26.8 (23.0) | 20.0 (13.4) | 25.4 (18.1) | 21.0 (11.6) | 59.2 (46.4) | 74.6 (40.8) | 81.6 (66.1) | 59.6 (47.3) |
| Age T_n_ (in years) | 14.7 (2.0) | 14.4 (2.1) | 14.5 (2.0) | 14.7 (1.8) | 16.4 (3.8) | 14.7 (2.7) | 15.8 (2.5) | 15.1 (2.0) |
| Population  Community  Marginalized  Clinical | 58.6  36.8  4.6 | 79.2  N/A  N/A | 94.1  N/A  N/A | 90.4  N/A  N/A | 83.7  N/A  N/A | 79.2  N/A  N/A | 72.0  N/A  N/A | 84.4  N/A  N/A |
| Shared informant | 92.0 | N/A | 70.6 | N/A | N/A | N/A | 60.0 | 65.6 |
| Adolescent boys | 49.3 (6.9) | 50.1 (5.6) | 50.5 (9.9) | 50.4 (5.5) | 43.2 (10.8) | 47.0 (6.7) | 46.4 (4.9) | 52.2 (16.6) |
| Racial-ethnic minority | 55.5 (40.1) | 16.4 (9.1) | 13.9 (10.6) | 17.3 (18.3) | 41.9 (39.8) | 45.0 (30.2) | 41.9 (30.8) | 38.2 (25.2) |
| Publication year | 2011 (5.4) | 2011 (6.8) | 2011 (7.0) | 2009 (7.3) | 2011 (6.0) | 2012 (6.8) | 2014 (3.9) | 2013 (5.1) |
| Journal impact factor | 2.1 (1.1) | 2.4 (1.4) | 2.1 (1.2) | 1.9 1.3) | 2.1 (0.9) | 3.2 (1.4) | 2.0 (1.0) | 2.1 (1.0) |
| Journal quartile | 2.3 (1.0) | 2.1 (1.2) | 2.2 (1.1) | 2.5 (1.2) | 1.8 (1.0) | 1.3 (0.8) | 2.1 (0.8) | 2.3 (0.9 |
| Publication status  Published  Unpublished | 97.1  2.9 | N/A  N/A | N/A  N/A | N/A  N/A | N/A  N/A | N/A  N/A | N/A  N/A | N/A  N/A |

Note. Time lag = time between measurements (in months), age T_n_ = adolescent age at first included measurement (in years), community = mostly racial-ethnic majority, non-clinical sample, marginalized = economically marginalized (e.g., low income) and/or racial-ethnic minority sample, clinical = clinical sample (including mental or physical disabilities), N/A = too few studies available.

# Table S6. *Model Fit Evaluation for Moderation Analyses*

|  | *Analyses* | | | | | | | | |
| --- | --- | --- | --- | --- | --- | --- | --- | --- | --- |
|  | *Three-level meta-regression – peer* | | | *Three-level meta-regression – romantic* | | | *MASEM – peer* | | |
| Moderator | Δχ^2^ | *df* | *p* | ΔΧ^2^ | *df* | *p* | Δχ^2^ | *df* | *p* |
| Time lag (in months) |  |  |  |  |  |  |  |  |  |
| support T_n_ 🡪 support T_n+1_ | 35.79 | 1 | <.001 | - | - | - | −272.68 | 2 | >.999 |
| support T_n_ 🡪 negative T_n+1_ | 1.33 | 1 | .249 | - | - | - | 13.54 | 2 | .001 |
| negative T_n_ 🡪 support T_n+1_ | 1.00 | 1 | .317 | 0.68 | 1 | .410 | −20.27 | 2 | >.999 |
| negative T_n_ 🡪 negative T_n+1_ | 15.20 | 1 | <.001 | 1.15 | 1 | .283 | 1.81 | 2 | .405 |
| Adolescent age (in years) |  |  |  |  |  |  |  |  |  |
| support T_n_ 🡪 support T_n+1_ | 6.11 | 2 | .047 | - | - | - | −35.22 | 23 | >.999 |
| support T_n_ 🡪 negative T_n+1_ | 0.08 | 1 | .783 | - | - | - | 7.125 | 2 | .028 |
| negative T_n_ 🡪 support T_n+1_ | <0.001 | 1 | .992 | 0.14 | 1 | .704 | 17.28 | 2 | <.001 |
| negative T_n_ 🡪 negative T_n+1_ | 3.94 | 1 | .047 | −2.05 | 2 | >.999 | 9.52 | 2 | .009 |
| Population: community |  |  |  |  |  |  |  |  |  |
| support T_n_ 🡪 support T_n+1_ | 0.05 | 1 | .816 | - | - | - | 0.64 | 2 | .726 |
| Population: marginalized |  |  |  |  |  |  |  |  |  |
| support T_n_ 🡪 support T_n+1_ | 0.10 | 1 | .756 | - | - | - | 0.27 | 2 | .872 |
| Population: clinical |  |  |  |  |  |  |  |  |  |
| support T_n_ 🡪 support T_n+1_ | 0.76 | 1 | .384 | - | - | - | 4.75 | 2 | .093 |
| Shared reporter |  |  |  |  |  |  |  |  |  |
| support T_n_ 🡪 support T_n+1_ | 3.89 | 1 | .048 | - | - | - | 5.18 | 2 | .075 |
| negative T_n_ 🡪 support T_n+1_ | 0.06 | 1 | .801 | 0.93 | 1 | .335 | 5.05 | 2 | .080 |
| negative T_n_ 🡪 negative T_n+1_ | - | - | - | 0.29 | 1 | .587 | - | - | - |

|  | *Analyses* | | | | | | | | |
| --- | --- | --- | --- | --- | --- | --- | --- | --- | --- |
|  | *Three-level meta-regression – peer* | | | *Three-level meta-regression – romantic* | | | *MASEM – peer* | | |
| Moderator | Δχ^2^ | *df* | *p* | ΔΧ^2^ | *df* | *p* | Δχ^2^ | *df* | *p* |
| Adolescent boys (%) |  |  |  |  |  |  |  |  |  |
| support T_n_ 🡪 support T_n+1_ | −20.52 | 13 | >.999 | - | - | - | 0.37 | 2 | .831 |
| support T_n_ 🡪 negative T_n+1_ | −9.17 | 7 | >.999 | - | - | - | 1.57 | 2 | .456 |
| negative T_n_ 🡪 support T_n+1_ | −7.40 | 7 | >.999 | −5.49 | 3 | >.999 | 2.53 | 2 | .282 |
| negative T_n_ 🡪 negative T_n+1_ | −6.18 | 7 | >.999 | −4.77 | 3 | >.999 | 5.65 | 2 | .059 |
| Racial-ethnic minority (%) |  |  |  |  |  |  |  |  |  |
| support T_n_ 🡪 support T_n+1_ | −63.19 | 37 | >.999 | - | - | - | −181.34 | 2 | >.999 |
| support T_n_ 🡪 negative T_n+1_ | −2.17 | 8 | >.999 | - | - | - | 6.05 | 2 | .049^[[2]](#footnote-2)^ |
| negative T_n_ 🡪 support T_n+1_ | −9.56 | 8 | >.999 | −5.49 | 3 | >.999 | −23.30 | 2 | >.999 |
| negative T_n_ 🡪 negative T_n+1_ | −8.50 | 13 | >.999 | −4.77 | 3 | >.999 | −29.18 | 2 | >.999 |
| Year of publication |  |  |  |  |  |  |  |  |  |
| support T_n_ 🡪 support T_n+1_ | 0.05 | 1 | .818 | - | - | - | 11.83 | .2 | .003 |
| support T_n_ 🡪 negative T_n+1_ | 0.22 | 1 | .642 | - | - | - | 4.74 | 2 | .093 |
| negative T_n_ 🡪 support T_n+1_ | 1.13 | 1 | .288 | 0.03 | 1 | .853 | 12.40 | 2 | .002 |
| negative T_n_ 🡪 negative T_n+1_ | 0.00 | 1 | .953 | 0.08 | 1 | .773 | 11.81 | 2 | .003 |
| Journal impact factor |  |  |  |  |  |  |  |  |  |
| support T_n_ 🡪 support T_n+1_ | −77.97 | 61 | >.999 | - | - | - | 9.74 | 2 | .008 |
| support T_n_ 🡪 negative T_n+1_ | 2.01 | 2 | .366 | - | - | - | 2.44 | 2 | .295 |
| negative T_n_ 🡪 support T_n+1_ | 0.16 | 1 | .689 | −6.11 | 4 | >.999 | 8.42 | 2 | .015 |
| negative T_n_ 🡪 negative T_n+1_ | −2.52 | 2 | >.999 | −16.45 | 6 | >.999 | 3.69 | 2 | .158 |
| Journal quartile |  |  |  |  |  |  |  |  |  |
| support T_n_ 🡪 support T_n+1_ | 7.88 | 6 | .247 | - | - | - | 21.76 | 2 | <.001 |

|  | *Analyses* | | | | | | | | |
| --- | --- | --- | --- | --- | --- | --- | --- | --- | --- |
|  | *Three-level meta-regression – peer* | | | *Three-level meta-regression – romantic* | | | *MASEM – peer* | | |
| Moderator | Δχ^2^ | *df* | *p* | ΔΧ^2^ | *df* | *p* | Δχ^2^ | *df* | *p* |
| support T_n_ 🡪 negative T_n+1_ | 1.58 | 2 | .453 | - | - | - | 0.94 | 2 | .625 |
| negative T_n_ 🡪 support T_n+1_ | 0.08. | 1 | .781 | −5.80 | 4 | >.999 | 2.62 | 2 | .270 |
| negative T_n_ 🡪 negative T_n+1_ | −1.80 | 2 | >.999 | −15.54 | 6 | >.999 | 2.93 | 2 | .231 |
| Publication status: unpublished |  |  |  |  |  |  |  |  |  |
| support T_n_ 🡪 support T_n+1_ | 0.86 | 1 | .355 | - | - | - | 4.51 | 2 | .105 |

*Note.* Time lag = time between measurements (in months), adolescent age = age at first included measurement (in years), community = mostly racial-ethnic majority, non-clinical sample, marginalized = economically marginalized (e.g., low income) and/or racial-ethnic minority sample, clinical = clinical sample (including mental or physical disabilities), − = not tested due to insufficient heterogeneity or too few studies.

# Table S7. *Summary of All Moderation Outcomes*

|  | *Three-level meta-regression – peer* | | | *Three-level meta-regression – romantic* | | | *MASEM – peer* | | |
| --- | --- | --- | --- | --- | --- | --- | --- | --- | --- |
| Moderator | β | *p* | 95% CI | β | *p* | 95% CI | CL_par>peer_  CL_peer>par_ | β | *p* |
| Time lag (in months) |  |  |  |  |  |  |  |  |  |
| support T_n_ 🡪 support T_n+1_ | −.002 | <.001 | [−.002;−.001] | - | - | - |  |  |  |
| support T_n_ 🡪 negative T_n+1_ |  |  |  | - | - | - |  | .001  .01 | .451  .067 |
| negative T_n_ 🡪 support T_n+1_ |  |  |  |  |  |  |  |  |  |
| negative T_n_ 🡪 negative T_n+1_ | −.002 | <.001 | [−.004;−.001] |  |  |  |  |  |  |
| Adolescent age (in years)^[[3]](#footnote-3)^ |  |  |  |  |  |  |  |  |  |
| support T_n_ 🡪 support T_n+1_ | .01 | .001 | [.004, .021] | - | - | - |  |  |  |
| support T_n_ 🡪 negative T_n+1_ |  |  |  | - | - | - |  | .01  .03 | .482  .042 |
| negative T_n_ 🡪 support T_n+1_ |  |  |  |  |  |  |  | .02  .03 | .003  .004 |
| negative T_n_ 🡪 negative T_n+1_ | .02 | .039 | [.00, .03] |  |  |  |  | −.01  −.04 | .542  .002 |
| Population: community |  |  |  |  |  |  |  |  |  |
| support T_n_ 🡪 support T_n+1_ |  |  |  | - | - | - |  |  |  |
| Population: marginalized |  |  |  |  |  |  |  |  |  |
| support T_n_ 🡪 support T_n+1_ |  |  |  | - | - | - |  |  |  |
| Population: clinical |  |  |  |  |  |  |  |  |  |
| support T_n_ 🡪 support T_n+1_ |  |  |  | - | - | - |  |  |  |

|  | *Three-level meta-regression – peer* | | | *Three-level meta-regression – romantic* | | | *MASEM – peer* | | |
| --- | --- | --- | --- | --- | --- | --- | --- | --- | --- |
| Moderator | β | *p* | 95% CI | β | *p* | 95% CI | CL_par>peer_  CL_peer>par_ | β | *p* |
| Shared informant |  |  |  |  |  |  |  |  |  |
| support T_n_ 🡪 support T_n+1_ | .10 | .046 | [.00; .20] | - | - | - |  |  |  |
| negative T_n_ 🡪 support T_n+1_ |  |  |  |  |  |  |  |  |  |
| Boys (%) |  |  |  |  |  |  |  |  |  |
| support T_n_ 🡪 support T_n+1_ |  |  |  | - | - | - |  |  |  |
| support T_n_ 🡪 negative T_n+1_ |  |  |  | - | - | - |  |  |  |
| negative T_n_ 🡪 support T_n+1_ |  |  |  |  |  |  |  |  |  |
| negative T_n_ 🡪 negative T_n+1_ |  |  |  |  |  |  |  |  |  |
| Racial-ethnic minority (%) |  |  |  |  |  |  |  |  |  |
| support T_n_ 🡪 support T_n+1_ |  |  |  | - | - | - |  |  |  |
| support T_n_ 🡪 negative T_n+1_ |  |  |  | - | - | - |  |  |  |
| negative T_n_ 🡪 support T_n+1_ |  |  |  |  |  |  |  |  |  |
| negative T_n_ 🡪 negative T_n+1_ |  |  |  |  |  |  |  |  |  |
| Year of publication |  |  |  |  |  |  |  |  |  |
| support T_n_ 🡪 support T_n+1_ |  |  |  | - | - | - |  | .003  .004 | .062 .019 |
| support T_n_ 🡪 negative T_n+1_ |  |  |  | - | - | - |  |  |  |
| negative T_n_ 🡪 support T_n+1_ |  |  |  |  |  |  |  | −.004  −.01 | .013  .005 |
| negative T_n_ 🡪 negative T_n+1_ |  |  |  |  |  |  |  | .001  .01 | .738  <.001 |

|  | *Three-level meta-regression – peer* | | | *Three-level meta-regression – romantic* | | | *MASEM – peer* | | |
| --- | --- | --- | --- | --- | --- | --- | --- | --- | --- |
| Moderator | β | *p* | 95% CI | β | *p* | 95% CI | CL_par>peer_  CL_peer>par_ | β | *p* |
| Journal impact factor |  |  |  |  |  |  |  |  |  |
| support T_n_ 🡪 support T_n+1_ |  |  |  | - | - | - |  | −.01  −.03 | .263  .009 |
| support T_n_ 🡪 negative T_n+1_ |  |  |  | - | - | - |  |  |  |
| negative T_n_ 🡪 support T_n+1_ |  |  |  |  |  |  |  | −.01  −.03 | .264  .009 |
| negative T_n_ 🡪 negative T_n+1_ |  |  |  |  |  |  |  | - | - |
| Journal quartile |  |  |  |  |  |  |  |  |  |
| support T_n_ 🡪 support T_n+1_ |  |  |  | - | - | - |  | .02  .04 | .029  <.001 |
| support T_n_ 🡪 negative T_n+1_ |  |  |  | - | - | - |  |  |  |
| negative T_n_ 🡪 support T_n+1_ |  |  |  |  |  |  |  |  |  |
| negative T_n_ 🡪 negative T_n+1_ |  |  |  |  |  |  |  |  |  |
| Publication status: unpublished |  |  |  |  |  |  |  |  |  |
| support T_n_ 🡪 support T_n+1_ |  |  |  | - | - | - |  |  |  |

*Note.* CI = lower and upper limits of confidence interval, CL_par>peer_ = cross-lagged path from parent T_n_ to peer T_n+1_, CL_peer>par_ = cross-lagged path from peer T_n_ to parent T_n+1_, empty cells = model fit did not significantly improve, − = not tested (no heterogeneity).

# Table S8. *Publication Bias*

|  | *Three-level analyses – Peer outcomes* | | | | |
| --- | --- | --- | --- | --- | --- |
|  | Egger’s test | | Three parameter selection | | |
|  | *z* | *p* | *r*_adjusted_ | χ^2^ | *p* |
| support T_n_ 🡪 support T_n+1_ | 0.78 | .435 | .21 | 0.20 | .652 |
| support T_n_ 🡪 negative T_n+1_ | −0.41 | .681 | - | - | - |
| negative T_n_ 🡪 support T_n+1_ | −0.86 | .388 | −.01 | 7.03 | .008 |
| negative T_n_ 🡪 negative T_n+1_ | −1.24 | .215 | .19 | 0.03 | .863 |
|  | *Three-level analyses – Romantic outcomes^[[4]](#footnote-4)^* | | | | |
| support T_n_ 🡪 support T_n+1_ | 1.25 | .210 | - | - | - |
| support T_n_ 🡪 negative T_n+1_ | −0.90 | .368 | - | - | - |
| negative T_n_ 🡪 support T_n+1_ | 2.47 | .013 | - | - | - |
| negative T_n_ 🡪 negative T_n+1_ | −0.01 | .995 | .16 | 0.77 | .380 |
|  | *MASEM analyses – Peer outcomes* | | | | |
| support T_n_ 🡪 support T_n+1_ | −0.94 | .349 | .19 | 3.44 | .064 |
| support T_n_ 🡪 negative T_n+1_ | −0.62 | .538 | −.12 | 0.29 | .592 |
| negative T_n_ 🡪 support T_n+1_ | −0.10 | .918 | −.06 | 0.65 | .419 |
| negative T_n_ 🡪 negative T_n+1_ | −0.78 | .435 | .20 | <.001 | .987 |

**
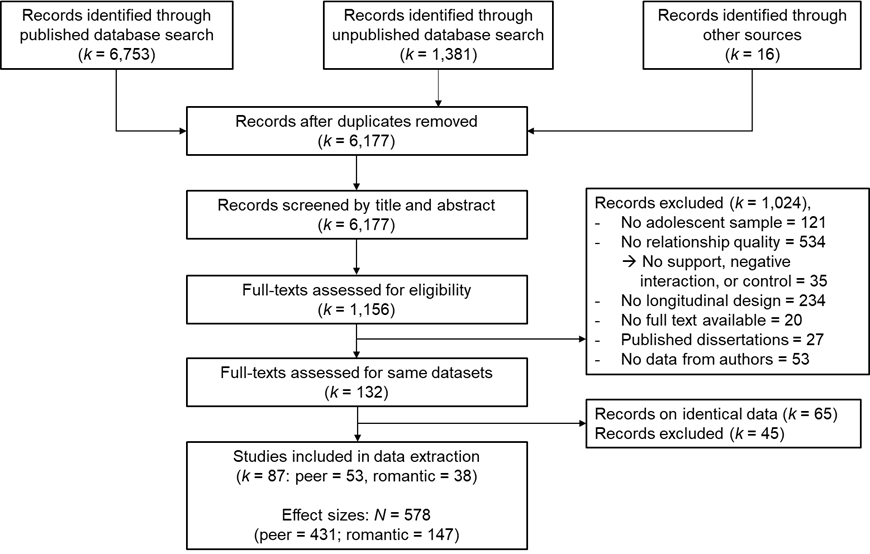
**

# *Figure S1.* PRISMA flow chart for study inclusion.

# Interpretation of forest plots (Figures S2-S9)

Forest plots regarding peer outcomes (see Figures S2-S5) indicate that particularly for associations across the same dimensions, study findings generally provide conclusive evidence, as indicated by mostly positive small-to-medium effects and many confidence intervals that do not include zero. For associations across different dimensions, study findings seem to exhibit similar conclusions from supportive parent-adolescent relationships to negative peer relationships, but less so from negative parent-adolescent relationships to supportive peer relationships, as indicated by mostly negligible effect sizes close to zero.

Forest plots regarding romantic outcomes (see Figures S6-S9) indicate that study findings generally point in the same direction for all associations with small-to-medium sized effects. While there was one study that also found medium-sized effects in the opposite direction for the association from negative parent-adolescent relationships to supportive romantic relationships, most of the confidence intervals included zero.


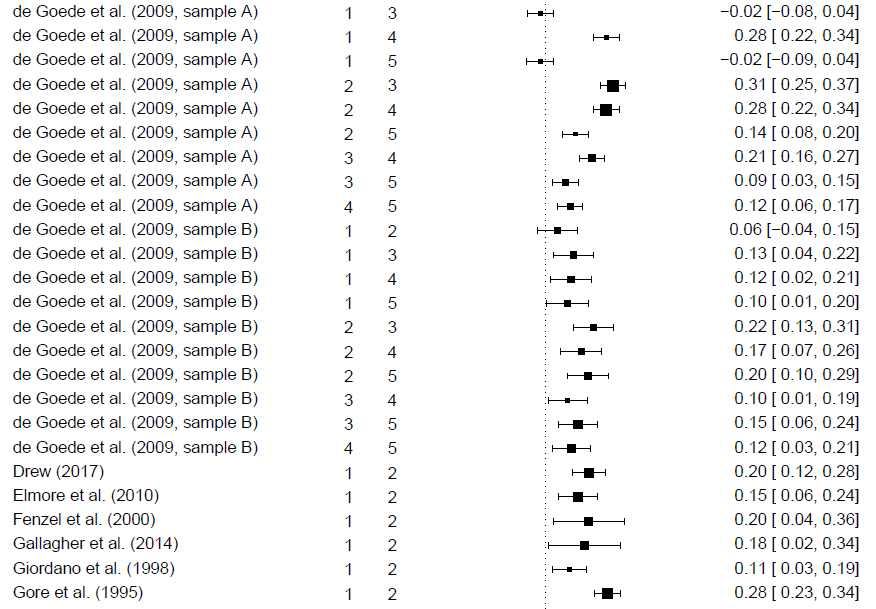

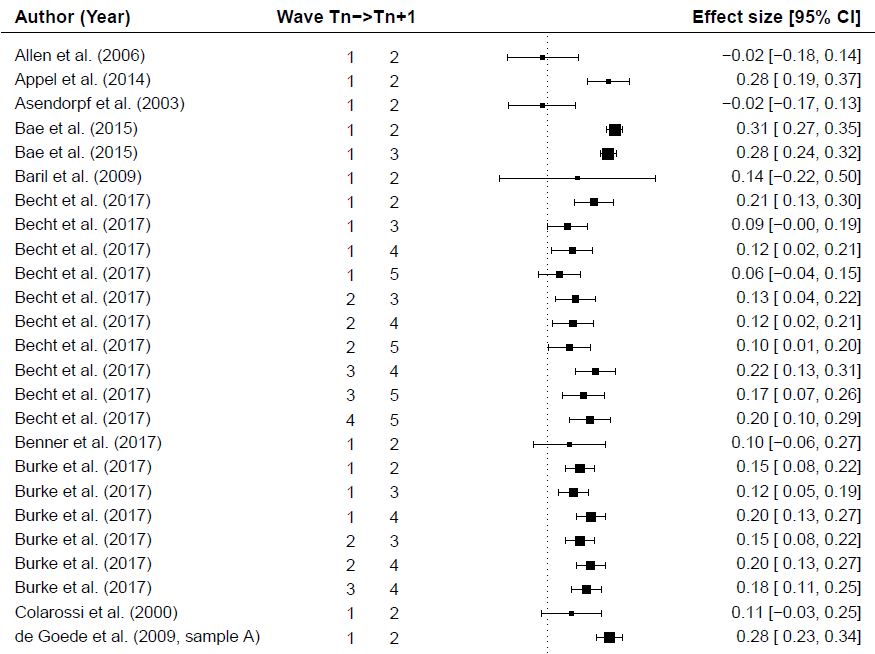

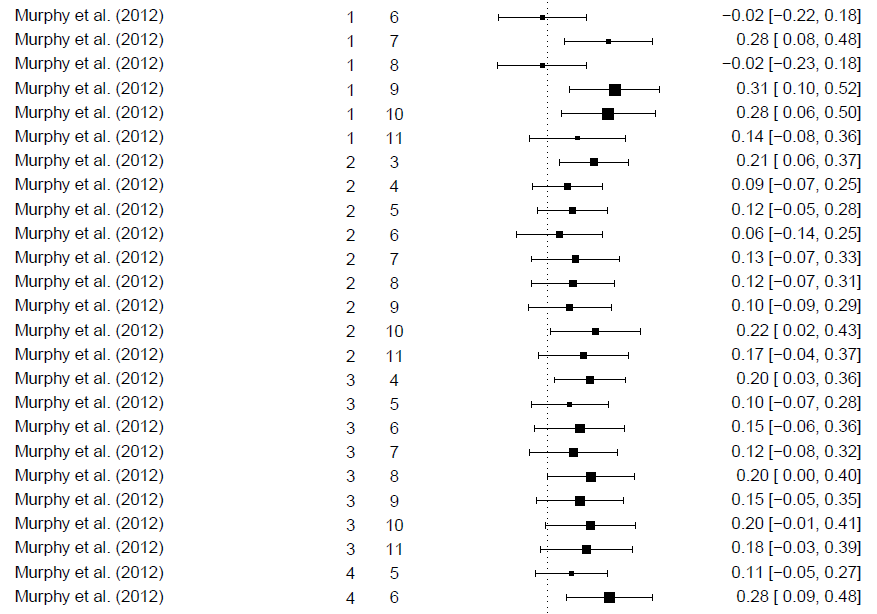

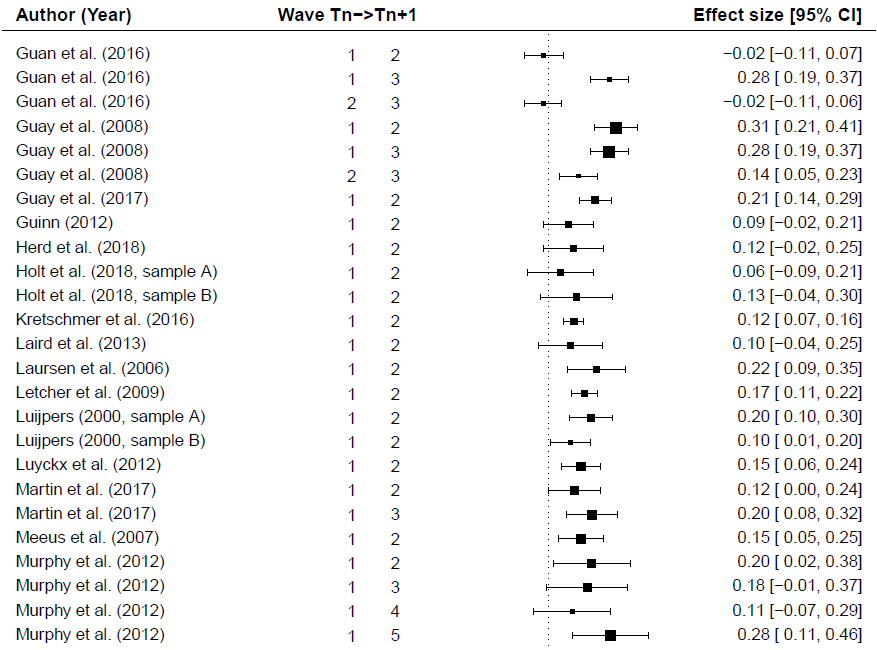

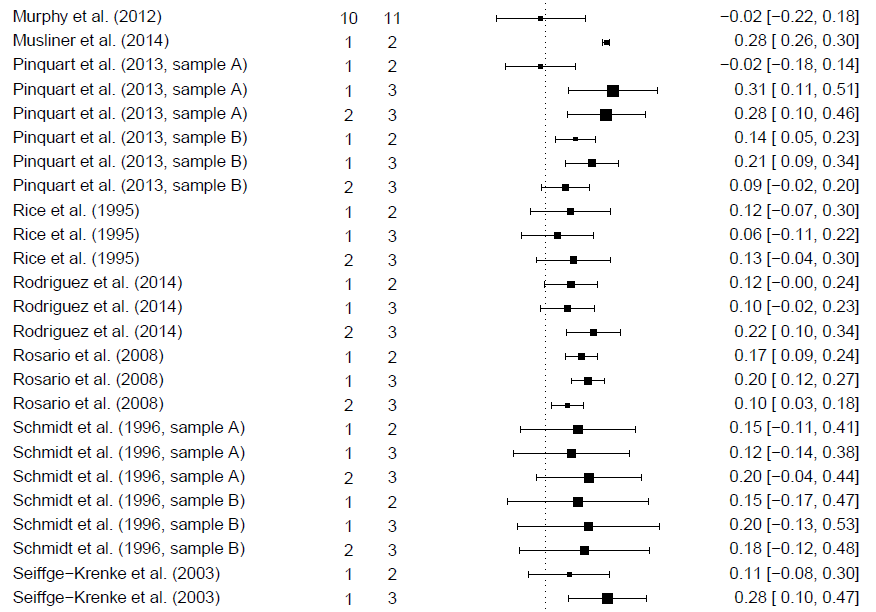

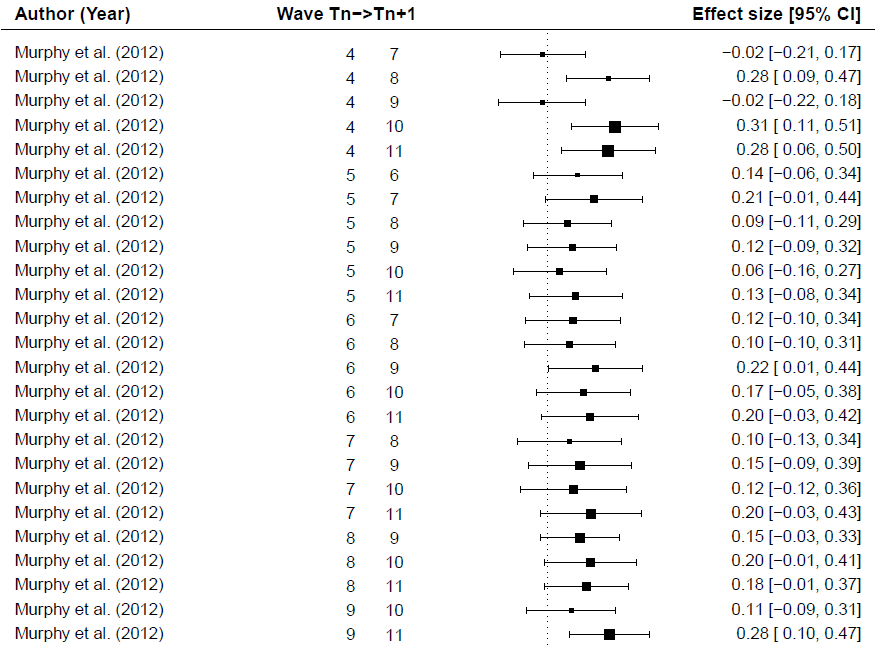


**
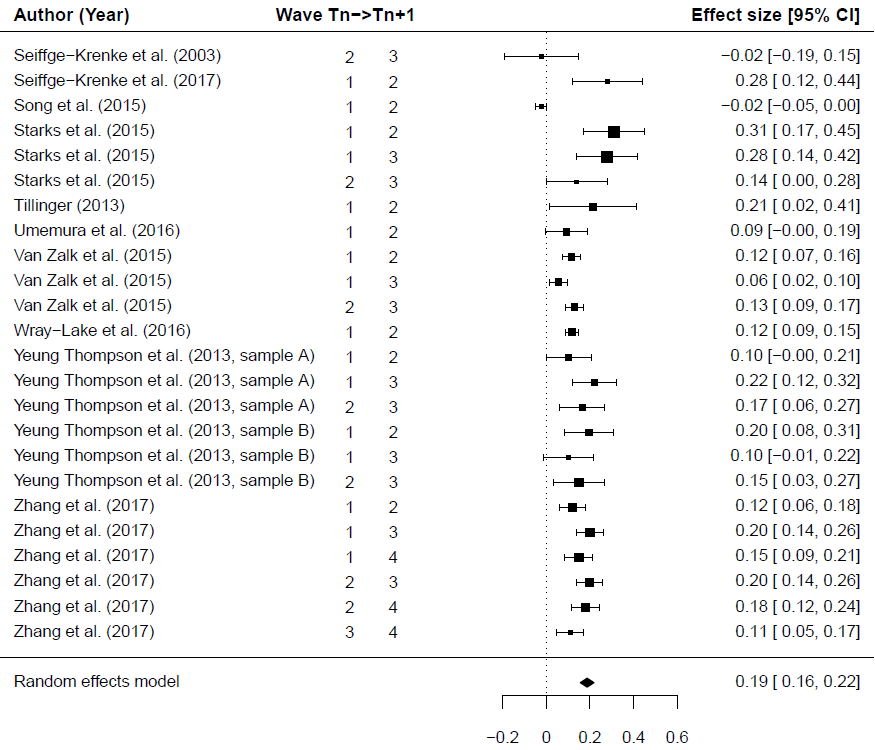
**

# *Figure S2.* Three-level random effects model for the associations between supportive parent-adolescent relationships and supportive peer relationships. Wave Tn = wave at parent-adolescent relationship; Tn+1 = wave at peer relationship; squared boxes depict weighted effect sizes with larger sample sizes receiving larger weights (indicated by the size of the box); whiskers depict confidence intervals.


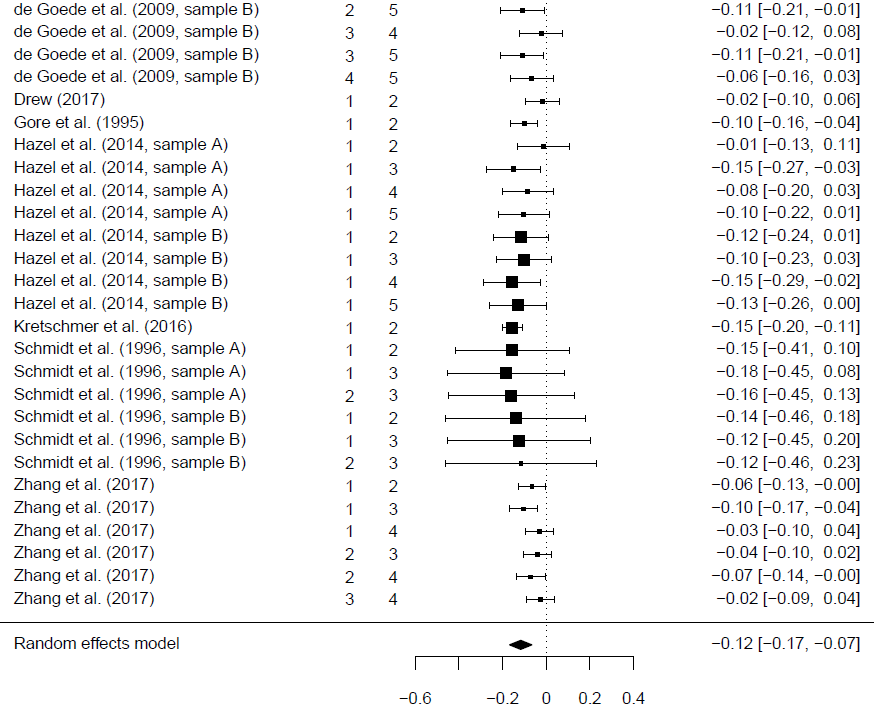

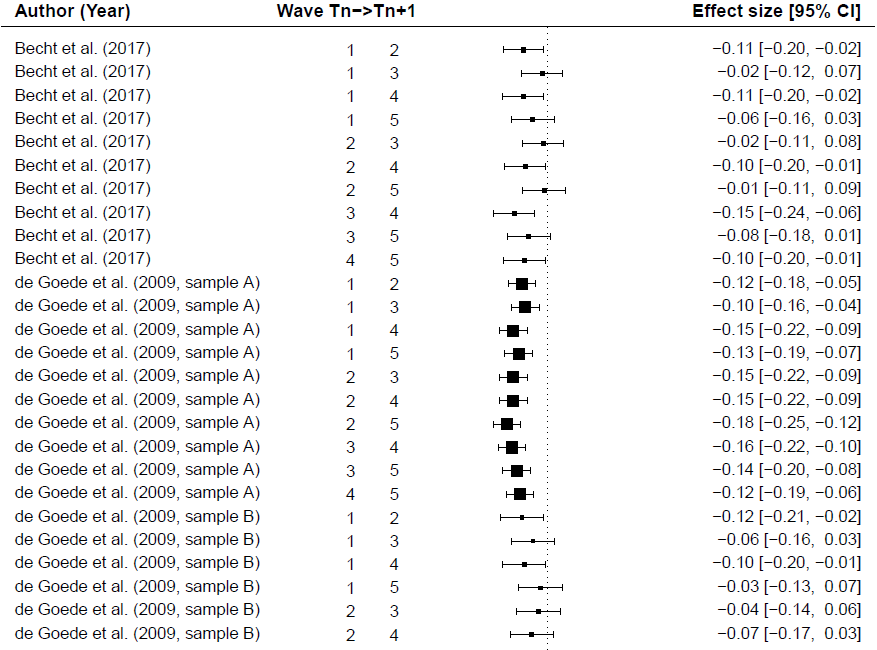


# *Figure S3.* Three-level random effects model for the associations between supportive parent-adolescent relationships and negative peer relationships. Wave Tn = wave at parent-adolescent relationship; Tn+1 = wave at peer relationship; squared boxes depict weighted effect sizes with larger sample sizes receiving larger weights (indicated by the size of the box); whiskers depict confidence intervals.


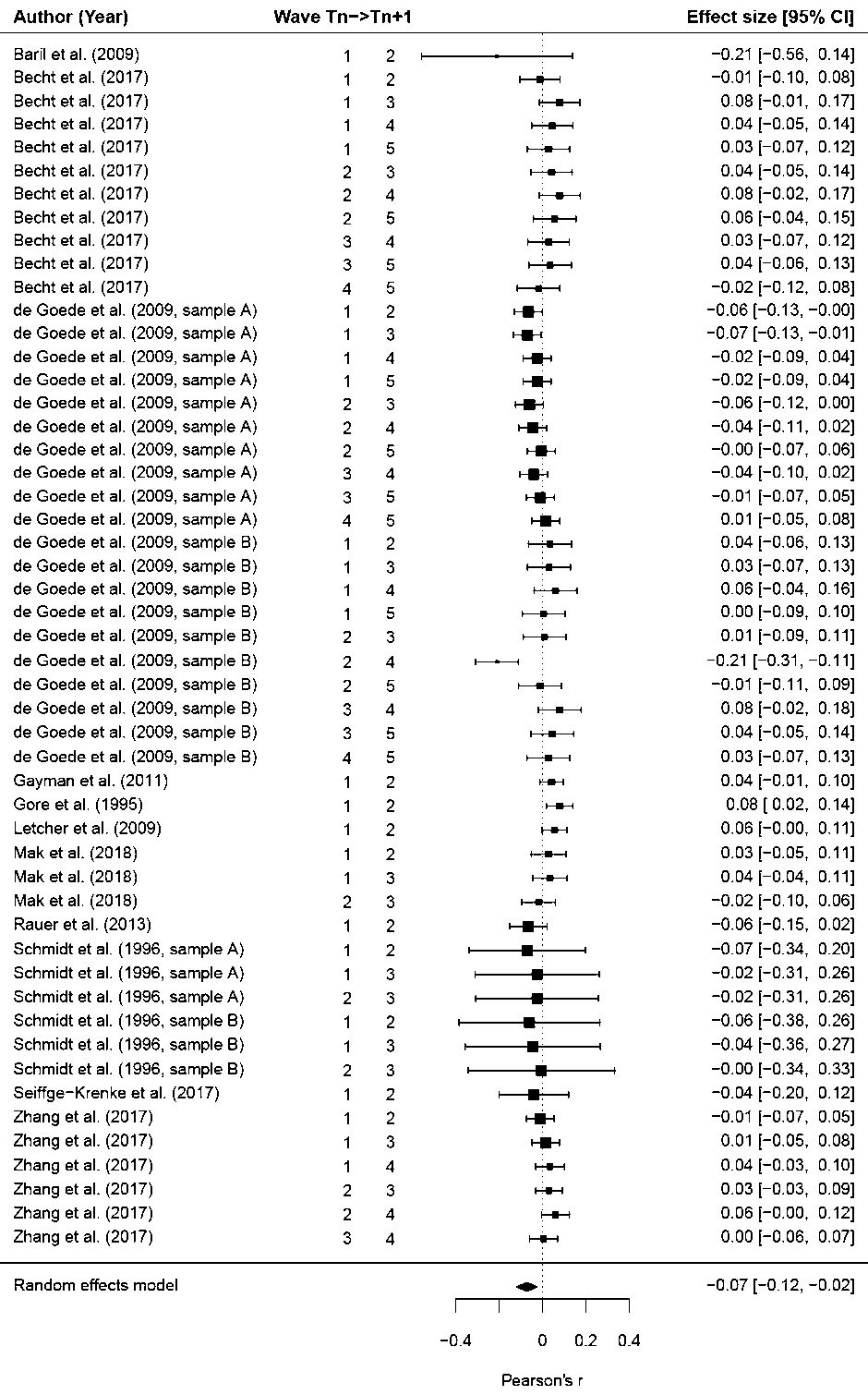


# *Figure S4.* Three-level random effects model for the associations between negative parent-adolescent relationships and supportive peer relationships. Wave Tn = wave at parent-adolescent relationship; Tn+1 = wave at peer relationship; squared boxes depict weighted effect sizes with larger sample sizes receiving larger weights (indicated by the size of the box); whiskers depict confidence intervals.


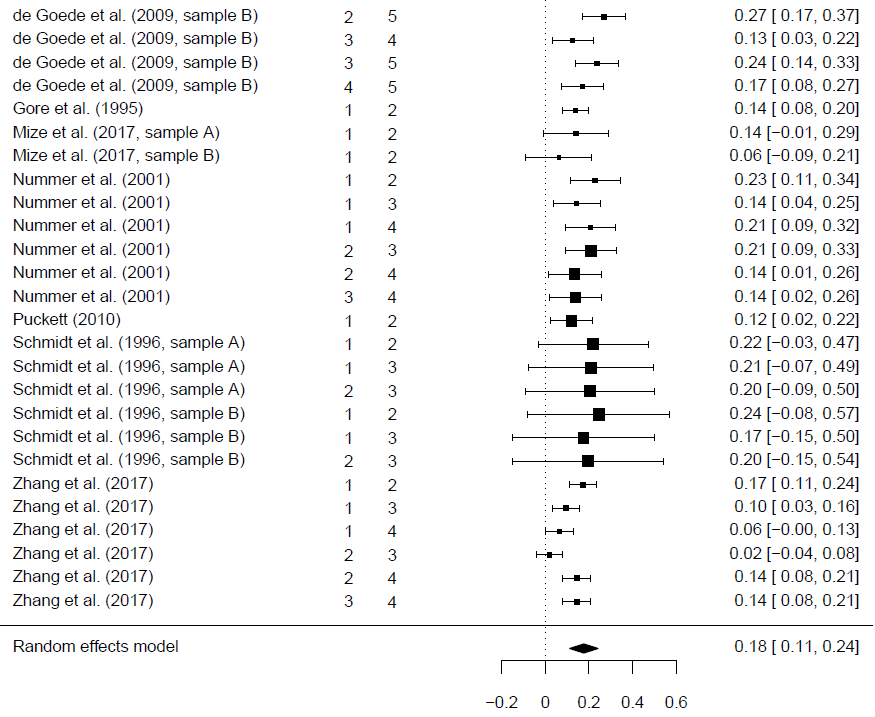

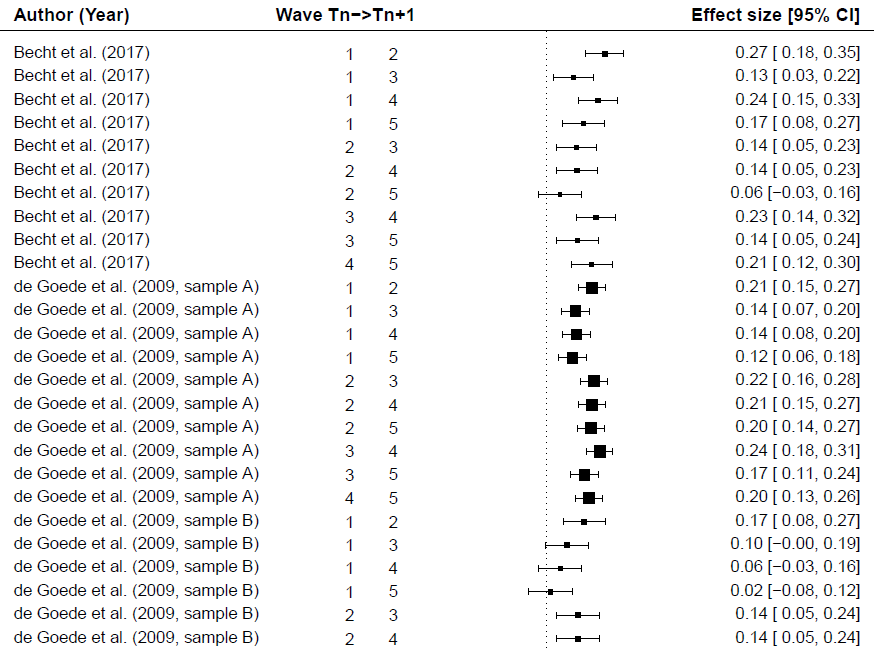


# *Figure S5.* Three-level random effects model for the associations between negative parent-adolescent relationships and negative peer relationships. Wave Tn = wave at parent-adolescent relationship; Tn+1 = wave at peer relationship; squared boxes depict weighted effect sizes with larger sample sizes receiving larger weights (indicated by the size of the box); whiskers depict confidence intervals.


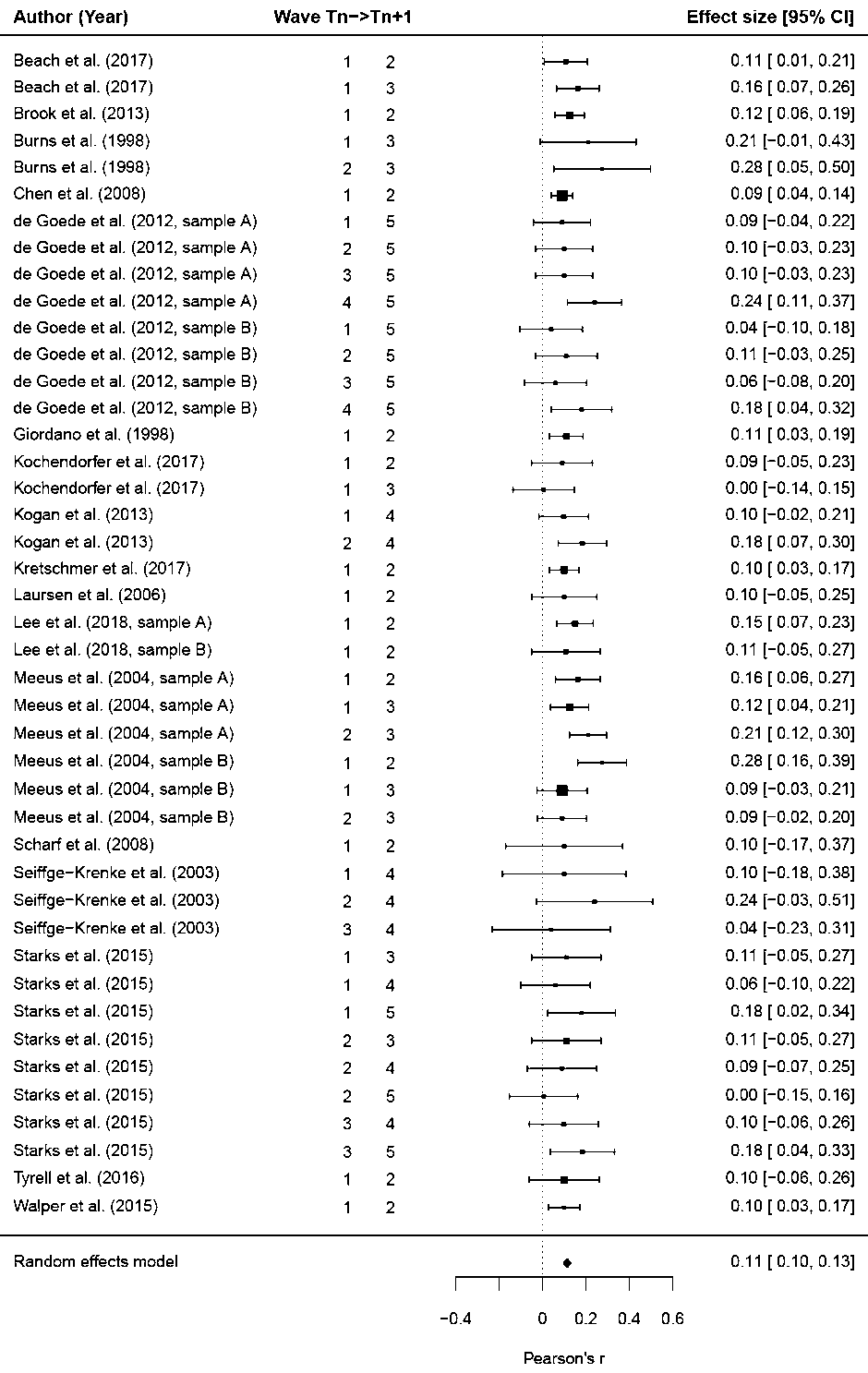


# *Figure S6.* Three-level random effects model for the associations between supportive parent-adolescent relationships and supportive romantic relationships. Wave Tn = wave at parent-adolescent relationship; Tn+1 = wave at romantic relationship; squared boxes depict weighted effect sizes with larger sample sizes receiving larger weights (indicated by the size of the box); whiskers depict confidence intervals.


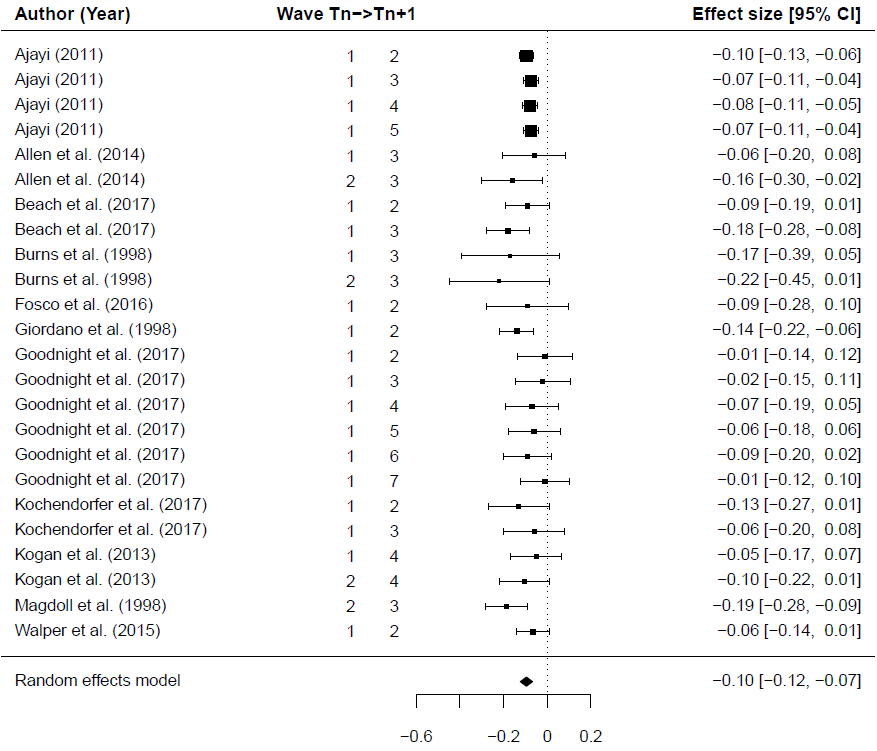


# *Figure S7.* Three-level random effects model for the associations between supportive parent-adolescent relationships and negative romantic relationships. Wave Tn = wave at parent-adolescent relationship; Tn+1 = wave at romantic relationship; squared boxes depict weighted effect sizes with larger sample sizes receiving larger weights (indicated by the size of the box); whiskers depict confidence intervals.


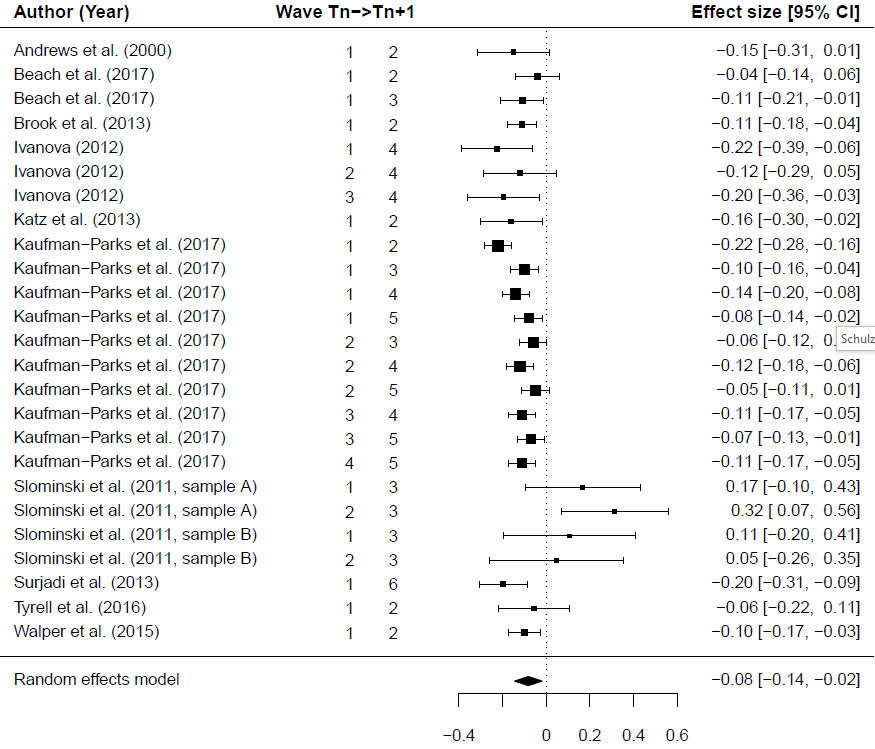


# *Figure S8.* Three-level random effects model for the associations between negative parent-adolescent relationships and supportive romantic relationships. Wave Tn = wave at parent-adolescent relationship; Tn+1 = wave at romantic relationship; squared boxes depict weighted effect sizes with larger sample sizes receiving larger weights (indicated by the size of the box); whiskers depict confidence intervals.


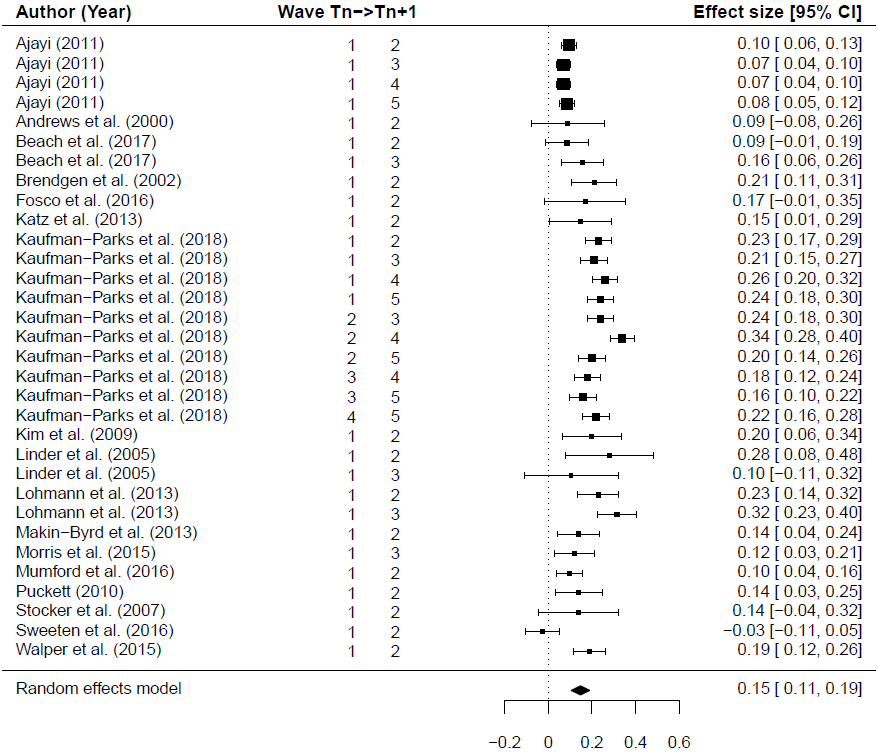


# *Figure S9.* Three-level random effects model for the associations between negative parent-adolescent relationships and negative romantic relationships. Wave Tn = wave at parent-adolescent relationship; Tn+1 = wave at romantic relationship; squared boxes depict weighted effect sizes with larger sample sizes receiving larger weights (indicated by the size of the box); whiskers depict confidence intervals.

1. Due to small variation on the third level of the analyses, the analysis resulted in problems estimating the standard errors. We therefore reran the analysis using the package metafor. [↑](#footnote-ref-1)
2. Due to small sample size and high model complexity, the analysis resulted in problems estimating reliable standard errors. We therefore do not report and interpret these results. None of the estimates were statistically significant (β_par🡪peer_ = −.004, *p* >.999; β_peer🡪par_ = −.002, *p* >.999). [↑](#footnote-ref-2)
3. Rerunning the moderation analyses without the estimated cases based on the country’s general age range per grade level yielded similar estimates and conclusions. [↑](#footnote-ref-3)
4. Due to low variation in effect sizes, we were unable to conduct three-parameter selection analyses for all but one association involving romantic relationships. However, recent research suggests that publication bias in relatively homogeneous datasets is weak at best (van Aert et al., 2019). [↑](#footnote-ref-4)
